# Exploring the Meta-regulon of the CRP/FNR Family of Global Transcriptional Regulators in a Partial-Nitritation Anammox Microbiome

**DOI:** 10.1128/mSystems.00906-21

**Published:** 2021-10-12

**Authors:** Natalie K. Beach, Kevin S. Myers, Brian R. Owen, Matt Seib, Timothy J. Donohue, Daniel R. Noguera

**Affiliations:** a Department of Civil and Environmental Engineering, University of Wisconsin-Madison, Madison, Wisconsin, USA; b Carollo Engineers, Inc., Broomfield, Colorado, USA; c Great Lakes Bioenergy Research Center, University of Wisconsin-Madison, Madison, Wisconsin, USA; d Wisconsin Energy Institute, University of Wisconsin-Madison, Madison, Wisconsin, USA; e Madison Metropolitan Sewerage District, Madison, Wisconsin, USA; f Department of Bacteriology, University of Wisconsin-Madison, Madison, Wisconsin, USA; g Carollo Engineers, Inc., San Diego, California, USA; Purdue University

**Keywords:** bioinformatics, metagenomics, metatranscriptomics, microbiome, transcription factors, wastewater treatment

## Abstract

Microorganisms must respond to environmental changes to survive, often by controlling transcription initiation. Intermittent aeration during wastewater treatment presents a cyclically changing environment to which microorganisms must react. We used an intermittently aerated bioreactor performing partial nitritation and anammox (PNA) to investigate how the microbiome responds to recurring change. Meta-transcriptomic analysis revealed a dramatic disconnect between the relative DNA abundance and gene expression within the metagenome-assembled genomes (MAGs) of community members, suggesting the importance of transcriptional regulation in this microbiome. To explore how community members responded to cyclic aeration via transcriptional regulation, we searched for homologs of the catabolite repressor protein/fumarate and nitrate reductase regulatory protein (CRP/FNR) family of transcription factors (TFs) within the MAGs. Using phylogenetic analyses, evaluation of sequence conservation in important amino acid residues, and prediction of genes regulated by TFs in the MAGs, we identified homologs of the oxygen-sensing FNR in *Nitrosomonas* and *Rhodocyclaceae*, nitrogen-sensing dissimilative nitrate respiration regulator that responds to nitrogen species (DNR) in *Rhodocyclaceae*, and nitrogen-sensing nitrite and nitric oxide reductase regulator that responds to nitrogen species (NnrR) in *Nitrospira* MAGs. Our data also predict that CRP/FNR homologs in *Ignavibacteria*, *Flavobacteriales*, and *Saprospiraceae* MAGs sense carbon availability. In addition, a CRP/FNR homolog in a Brocadia MAG was most closely related to CRP TFs known to sense carbon sources in well-studied organisms. However, we predict that in autotrophic Brocadia, this TF most likely regulates a diverse set of functions, including a response to stress during the cyclic aerobic/anoxic conditions. Overall, this analysis allowed us to define a meta-regulon of the PNA microbiome that explains functions and interactions of the most active community members.

**IMPORTANCE** Microbiomes are important contributors to many ecosystems, including ones where nutrient cycling is stimulated by aeration control. Optimizing cyclic aeration helps reduce energy needs and maximize microbiome performance during wastewater treatment; however, little is known about how most microbial community members respond to these alternating conditions. We defined the meta-regulon of a PNA microbiome by combining existing knowledge of how the CRP/FNR family of bacterial TFs respond to stimuli, with metatranscriptomic analyses to characterize gene expression changes during aeration cycles. Our results indicated that, for some members of the community, prior knowledge is sufficient for high-confidence assignments of TF function, whereas other community members have CRP/FNR TFs for which inferences of function are limited by lack of prior knowledge. This study provides a framework to begin elucidating meta-regulons in microbiomes, where pure cultures are not available for traditional transcriptional regulation studies. Defining the meta-regulon can help in optimizing microbiome performance.

## INTRODUCTION

In natural ecosystems, microbiomes often experience cyclical variations in environmental conditions such as presence or absence of light (e.g., day/night cycles), warm and cold temperatures (e.g., day/night, summer/winter), and presence or absence of oxygen (e.g., low tide/high tide). Microbiomes are used in advanced wastewater treatment processes for the removal of nutrients, where controlling the presence and absence of oxygen creates a dynamic environment that allows bacteria to carry out important functions such as enhanced biological phosphorus removal, nitrification, and denitrification (1, 2). Likewise, intermittent aeration enables the use of microbiomes enriched with anaerobic ammonium-oxidizing bacteria (anammox) to treat sidestream wastewaters containing high concentrations of ammonium (3). We are interested in the microbial response to cycles of low dissolved oxygen (DO) and anoxic conditions because oxygen delivery accounts for a large fraction of the electricity required to operate a wastewater treatment process. Reducing oxygen requirements is one attractive strategy to decrease energy use during wastewater treatment (4, 5). In addition, anammox processes have the potential to further reduce energy requirements in wastewater treatment plants (6).

Microbial adaptation is critical for survival in changing environments like those experienced by wastewater treatment microbiomes. Transcription factors (TFs) often regulate this adaptation by binding to DNA and increasing or decreasing transcription initiation in response to specific environmental stimuli. Examples of microbial adaptation to changing environments by transcriptional regulation include the expression of genes that encode proteins involved in alternative metabolic pathways in response to changes in carbon source, oxygen, or alternative electron acceptors (7). Bacterial TFs can regulate genes within hundreds of operons or only those within a single operon (7–9), so the number of genes that are directly controlled by these proteins (its regulon) can vary in size.

One particular TF, the fumarate and nitrate reductase regulatory protein (FNR), is among the most widely studied global regulators, activating and repressing transcription of a wide variety of functional genes. FNR is widely conserved across bacteria and is often required for the shift between aerobic and anaerobic metabolism (10). FNR belongs to the catabolite repressor protein (CRP; also known as cAMP receptor protein)/FNR family of TFs, which plays an important role across bacteria and generally shares a conserved DNA binding site (11). Like FNR, CRP is well studied and widely conserved across bacteria and responds to variations in glucose availability via changes in the concentration of cyclic AMP (12). Besides CRP and FNR, the CRP/FNR family of proteins contains other well-studied TFs that respond to a variety of environmental stimuli, including dissimilative nitrate respiration regulator that responds to nitrogen species (DNR), anaerobic regulator of arginine deiminase and nitrate reductase that responds to oxygen and nitrogen species (ANR), nitrite and nitric oxide reductase regulator that responds to nitrogen species (NnrR), and maltose regulator that responds to changes in carbohydrate carbon source (MalR) (11, 13–17).

Although the environmental stimuli and the function of genes regulated by CRP/FNR family TFs have been well studied in pure cultures of several bacterial species, little work has been done to study how homologs of these proteins may function in a microbiome. Given the conservation of members of the CRP/FNR family across bacteria (11, 16), we reasoned that potential members of the CRP/FNR family may simultaneously control the activity of multiple organisms within a microbiome exposed to cycles of low-DO/anoxic conditions as well as various concentrations of nitrogen species. Elucidating a microbiome-level regulon (i.e., a meta-regulon) from members of the CRP/FNR family of TFs could provide further insight into the function of the often unculturable key community members and their interactions within the microbiome.

This study utilized a metagenomic and metatranscriptomic approach to identify the predicted CRP/FNR family meta-regulon in an intermittently aerated deammonification bioreactor performing partial nitritation and anammox (PNA). Out of 43 high-quality metagenome-assembled genomes (MAGs) reconstructed from the microbiome, CRP/FNR homologs were detected in 29, including 11 of the top 16 MAGs as determined by RNA abundance in metatranscriptomic data sets. We predicted potential DNA binding sites for CRP/FNR family TFs and correlated these predictions with metatranscriptomic changes to predict which genes were regulated during cyclic bioreactor conditions. This analysis provides a first glimpse of how a microbiome collectively responds to external environmental factors. This information, and the methodology described here, both improves our understanding of uncultured microorganisms and their function in changing environmental conditions and generates models that could enhance our capacity to harness microbiomes for specific functions, such as nitrogen removal from wastewater.

## RESULTS

### Performance of PNA bioreactor with intermittent aeration.

**FIG 1:**
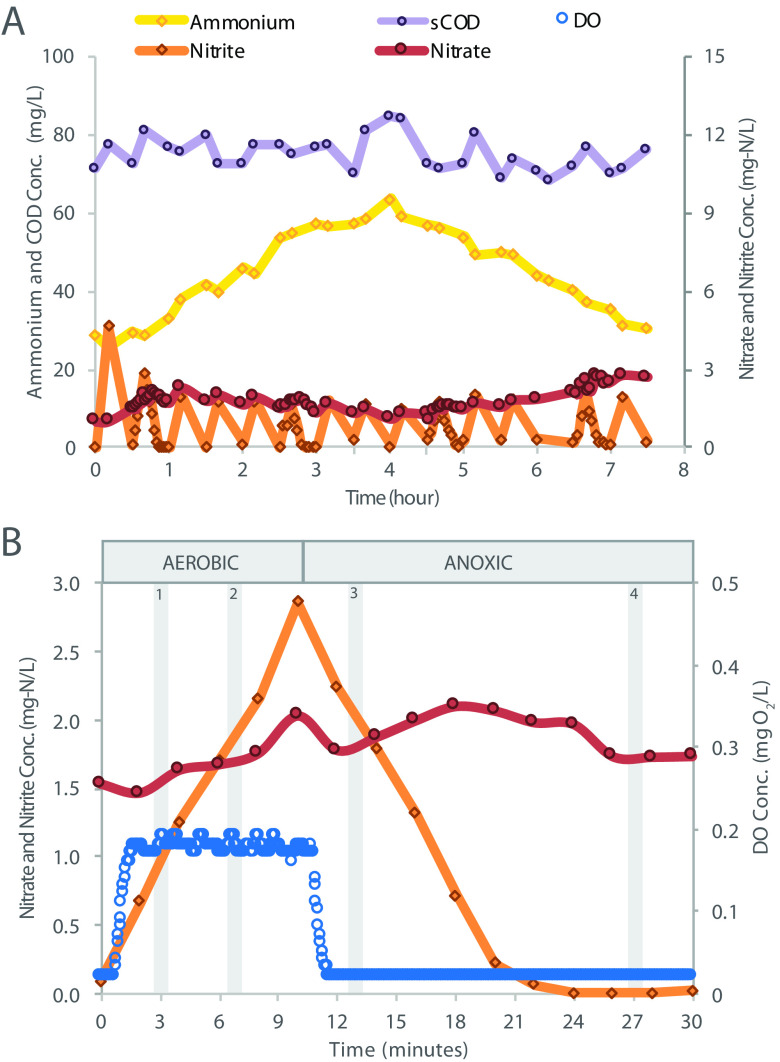
Typical bioreactor performance during a period of stable operation. (A) Typical chemical profile during the fill and react phases of a single 8-h cycle of operation; all measurements are from filtered extracellular samples collected from the bioreactor. (B) Closeup of a 30-min time interval, defined by 10-min aerated (aerobic) and 20-min unaerated (anoxic) periods. Samples were collected for RNA sequencing at 3 min and 7 min into the aerated period, indicated by “1” and “2” shaded regions, and at 13 min and 27 min in the unaerated period, indicated by “3” and “4” shaded regions.

The performance of the PNA bioreactor was evaluated for 3 years. After a startup period with unstable performance that lasted for nearly 1 year, the reactor stabilized. A summary of reactor performance during stable operation is shown in Table 1, and representative nutrient profiles are shown in Fig. 1. The reactor was fed ammonium-rich reject water from a struvite recovery process operated at the Nine Springs wastewater treatment plant (Madison, WI). The chemical composition of the feed varied from week to week depending on full-scale plant operations and performance of upstream processes, with average ammonium concentration of 198 ± 61 mg NH_4_^+^-N/liter and variable organic carbon concentrations ranging primarily between 100 and 400 mg chemical oxygen demand (COD)/liter, with a few weeks exceeding 1,000 mg COD/liter (Table 1). During the longest period of stable operation (∼560 days), the average total inorganic nitrogen and ammonium removal were 72% and 83%, respectively. During the first 4-h of a typical 8-h cycle, the reactor was slowly filled with reject water, and ammonium accumulated (Fig. 1A). In the remaining 3.5-h, ammonium concentrations declined. Nitrate and soluble COD remained almost constant throughout the cycle, while nitrite concentrations fluctuated according to aerated (nitrite increased) and unaerated (nitrite decreased) periods. During normal operation, the DO remained below the control set point of 0.2 mg O_2_/liter (Fig. 1B). Total suspended solids (TSS) and volatile suspended solids (VSS) within the bioreactor averaged 1,570 ± 550 mg TSS/liter and 1,200 ± 440 mg VSS/liter, respectively (Table 1).

**TABLE 1:** Summary of bioreactor properties, feed, and effluent composition over 3 years of operation

Characteristic^*a*^	No. of samples	Max	Min	Median	Avg	SD
Reactor properties						
TSS (mg/liter)	259	3,420	440	1,520	1,570	550
VSS (mg/liter)	259	3,010	290	1,130	1,200	440
pH	471	8.2	6.3	7.48	7.46	0.28
Temp (°C)	471	36.9	21	33	32.8	1.94
Feed composition						
Ammonium-N (mg N/liter)	223	368	80.1	186	198	61.5
Nitrate (mg N/liter)	220	6.82	0	0.20	0.58	1.10
Nitrite (mg N/liter)	220	13.1	0	0.03	0.42	1.62
Total COD (unfiltered) (mg O_2_/liter)	210	2,290	75.5	394	421	240
Soluble COD (filtered) (mg O_2_/liter)	191	2,150	63.6	220	241	160
pH	38	8.66	7.74	8.0	8.1	0.20
Effluent composition						
Ammonium-N (mg N/liter)	463	289	0.10	31.2	49.7	57.5
Nitrate (mg N/liter)	461	83.6	0	21.2	25.0	20.9
Nitrite (mg N/liter)	461	172	0	0.09	4.70	18.8
Soluble COD (filtered) (mg O_2_/liter)	410	500	6.6	67.2	76.3	37.7
Performance summary						
Total inorganic N removed (%)	220	99	0	60	58	23
Ammonium removed (%)	223	100	0	82	69	32

a TSS, total suspended solids; VSS, volatile suspended solids; ammonium-N, NH_4_^+^ plus NH_3_; nitrate, NO_3_^−^; nitrite, NO_2_^−^; COD; chemical oxygen demand.

### Metagenomic and transcriptomic sequencing, binning, and quality control.

To evaluate the composition of the bioreactor microbiome, we collected and extracted DNA from 4 days of operation (days 77, 231, 350, and 454) for metagenomic sequencing. DNA from each sample was sequenced twice, resulting in eight separate metagenomic DNA read data sets. Of ∼206 million metagenomic reads, ∼201 million passed quality control criteria and were used as input for one metagenomic coassembly (Table S1 in the supplemental material) that generated 2,637 scaffolds. From this coassembly, we constructed 43 highly complete (>90%), low-contamination (<6%) MAGs using a combination of automatic and manual binning and curation (Table S2).

10.1128/mSystems.00906-21.3 TABLE S1 DNA and mRNA alignment statistics. Download Table S1, XLSX file, 0.03 MB. Copyright © 2021 Beach et al. 2021 Beach et al. https://creativecommons.org/licenses/by/4.0/ This content is distributed under the terms of the Creative Commons Attribution 4.0 International license.

10.1128/mSystems.00906-21.4 TABLE S2 Metagenome and metatranscriptome statistics. Download Table S2, XLSX file, 0.02 MB. Copyright © 2021 Beach et al. 2021 Beach et al. https://creativecommons.org/licenses/by/4.0/ This content is distributed under the terms of the Creative Commons Attribution 4.0 International license.

Twenty-four samples were collected at day 454 of bioreactor operation for metatranscriptomic analysis (these reads are referred to here as mRNA reads). Approximately 306 million mRNA reads aligned to the 43 MAGs (out of ∼411 million mRNA reads that mapped to the assembly) (Tables S1, S2, and S3), indicating that the 43 MAGs captured a large fraction of the metatranscriptome (74%). Further, out of the mRNA reads mapping to the 43 high-quality MAGs, 95% of them mapped to only 16 MAGs, which we define here as the most active community members present in the reactor on day 454 (Table 2).

**TABLE 2:** Genome statistics of the top 16 MAGs with the highest relative gene expression recovered from the PNA bioreactor

MAG ID	Taxonomy	Completeness (%)	Contamination (%)	Genome size (Mb)	No. of scaffolds	GC content (%)	No. of predicted genes	Relative DNA abundance (%)	Relative mRNA abundance (%)
IGV_58	*Ignavibacteria* bacterium	95.08	0	2.41	15	37.7	2,131	32.3	39.6
AMX_44	*Brocadia* sp.	90.11	1.65	2.79	111	44.9	2,553	1.3	19.7
NSM_48	Nitrosomonas europaea	96.66	0.62	2.64	63	50.7	2,467	2.1	16.8
NSP_46	*Nitrospira* sp.	96.76	5.23	3.79	39	60.4	3,606	1.3	3.1
ANR_43	*Anaerolineales* bacterium	88.18	3.27	3.8	262	59.2	3,476	1.4	2.5
RDC_57	*Rhodocyclaceae* bacterium	94.75	0.93	3.03	60	66.6	3,016	7.2	2.1
FLB_49	*Flavobacteriales* bacterium	100	0.54	3.09	27	36.1	2,711	1.4	2.0
RDC_54R	*Rhodocyclaceae* bacterium	92.13	0.93	2.84	41	66.6	2,820	4.2	1.8
XAM_3	*Xanthomonadales* bacterium	97.73	1.53	3.83	81	67.9	3,293	2.6	1.8
IGV_68R	*Ignavibacteriaceae* bacterium	94.41	0.56	3.44	17	42.1	2,712	0.5	1.3
SPS_50R	*Saprospiraceae* bacterium	97.36	0.5	3.66	73	49.6	2,868	1.4	1.1
ANR_55	*Anaerolineales* bacterium	93.64	0.91	4.06	62	55.9	3,695	4.3	1.0
STB_2	*Steroidobacteraceae* bacterium	90.18	1.55	3.57	80	67.6	3,367	2.2	1.0
ANR_56R	*Anaerolineales* bacterium	92.73	0.18	3.78	16	53.7	3,541	2.2	0.8
NSM_41	*Nitrosomonas* sp.	96.86	0.48	3.1	75	49.3	2,996	1.6	0.8
BRB_32R	*Bryobacteraceae* bacterium	91.23	1.32	5.83	110	62.1	4,924	0.4	0.8

10.1128/mSystems.00906-21.5 TABLE S3 Metatranscriptomic linear RPKM values. Download Table S3, XLSX file, 18.4 MB. Copyright © 2021 Beach et al. 2021 Beach et al. https://creativecommons.org/licenses/by/4.0/ This content is distributed under the terms of the Creative Commons Attribution 4.0 International license.

### Phylogenetic analysis and placement of MAGs.

**FIG 2:**
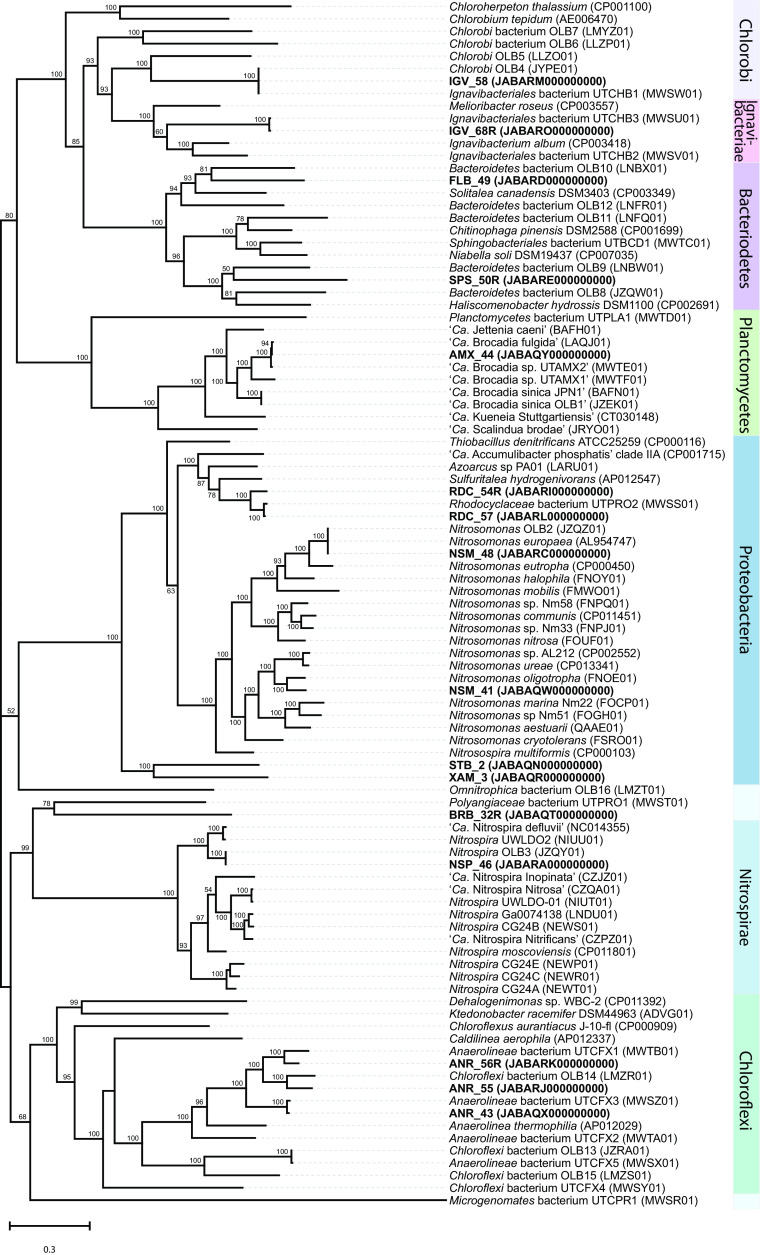
Phylogenetic tree generated from genome alignment of the 16 most active MAGs (Table 2). The tree includes MAGs recovered from this study (bold) and closely related genomes downloaded from the NCBI genome repository. Accession numbers for each genome are provided in parentheses. Bootstrap values are shown at the nodes where the value was greater than 50. The tree was constructed using RAxML based on a set of 37 concatenated universal single-copy marker genes.

We compared the phylogenetic relationships of the 16 MAGs representing the most active community members with published genomes and MAGs spanning across 7 different phyla (Fig. 2). This phylogenetic tree was constructed based on concatenated protein sequences of 37 conserved bacterial single-copy marker genes (18). The tree topology shows that many of the most active community members in this bioreactor clustered closely with published genomes from other deammonification bioreactors. Six of the MAGs (IGV_58, FLB_49, SPS_50R, NSM_48, NSP_46, and ANR_55) grouped closely with genomes recovered from a full-scale granular PNA reactor (OLB in genome names in Fig. 2) in Olburgen, The Netherlands (19). Likewise, six MAGs (IGV_58, IGV_68R, AMX_44, RDC_57, ANR_56R, and ANR_43) clustered closely with genomes recovered from a laboratory-scale anammox bioreactor (UT_ in genome names in Fig. 2), which was initially inoculated with biomass enriched with anammox from the City College of New York (6). Other notable groupings are NSM_48 with the ammonia-oxidizing bacteria (AOB) Nitrosomonas europaea (GenBank accession no. AL954747), which was also the main AOB in the Olburgen PNA bioreactor (19), and NSM_41 clustering with Nitrosomonas oligotropha, an AOB lineage reported to have high affinity for oxygen (20–22). In addition, NSP_46 clustered with the nitrite-oxidizing bacteria (NOB) ‘*Candidatus* Nitrospira defluvii’ (GenBank accession no. NC_014355.1), which has been reported in wastewater treatment systems operated under low-DO conditions (23, 24). The clustering of NSP_46 with ‘*Ca*. Nitrospira defluvii’ indicates that NSP_46 is a canonical NOB and not a complete ammonia-oxidizing (comammox) *Nitrospira*, since representatives of comammox (‘*Candidatus* Nitrospira inopinata,’ ‘*Candidatus* Nitrospira nitrosa,’ and ‘*Candidatus* Nitrospira nitrificans’) formed a different cluster within *Nitrospirae* (Fig. 2).

### Microbial community abundance and gene expression.

**FIG 3:**
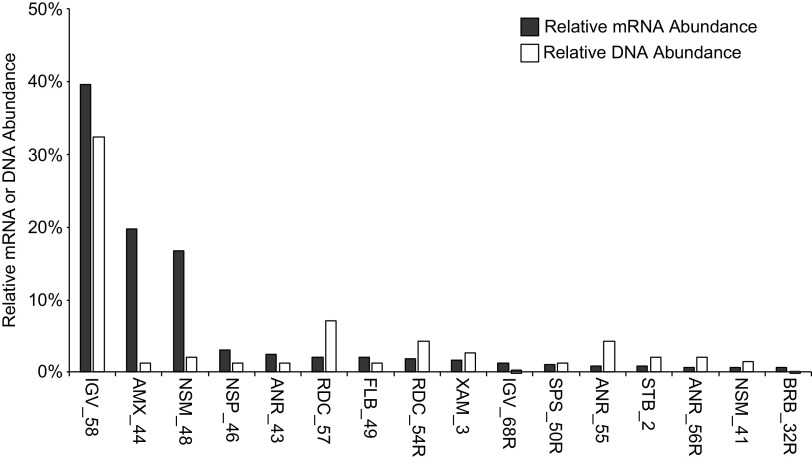
Relative DNA (white bars) and mRNA abundance (gray bars) of the most active 16 MAGs (Table 2). DNA abundances were determined by the number of DNA reads mapped to each MAG, normalized by the corresponding genome size. mRNA abundances were determined by the number of mRNA reads that mapped to each MAG, normalized by the corresponding genome size. Relative abundances were then determined dividing by the total number of DNA or mRNA reads for all 43 high-quality MAGs, respectively.

To calculate relative DNA and mRNA abundance (also referred to as relative gene expression), we normalized the respective nucleic acid read counts for each MAG by its predicted genome size and then divided this quantity by the total DNA or mRNA read counts for all 43 MAGs, respectively. The direct comparison of these two metrics revealed that relative DNA abundance and gene expression did not always correlate for each MAG. For example, MAGs associated with nitrogen cycling such as NSM_48 (*Nitrosomonas*), AMX_44 (*Brocadia*), and NSP_46 (*Nitrospira*), accounted for just 2.1%, 1.3%, and 1.3% relative DNA abundance (Fig. 3, white bars), respectively. However, these MAGs accounted for approximately 20%, 17%, and 3% of the relative gene expression, respectively (Fig. 3, gray bars). An *Ignavibacteria* (IGV_58) MAG dominated in both categories, with relative DNA abundance and relative gene expression at ∼32% and ∼40%, respectively. Other MAGs within the 16 most active groups had variable levels of relative DNA abundance (from 0.4% to 7.2%) but less than 3% relative gene expression.

### Phylogenetic analysis of CRP/FNR family TFs identified in the PNA microbiome.

**FIG 4:**
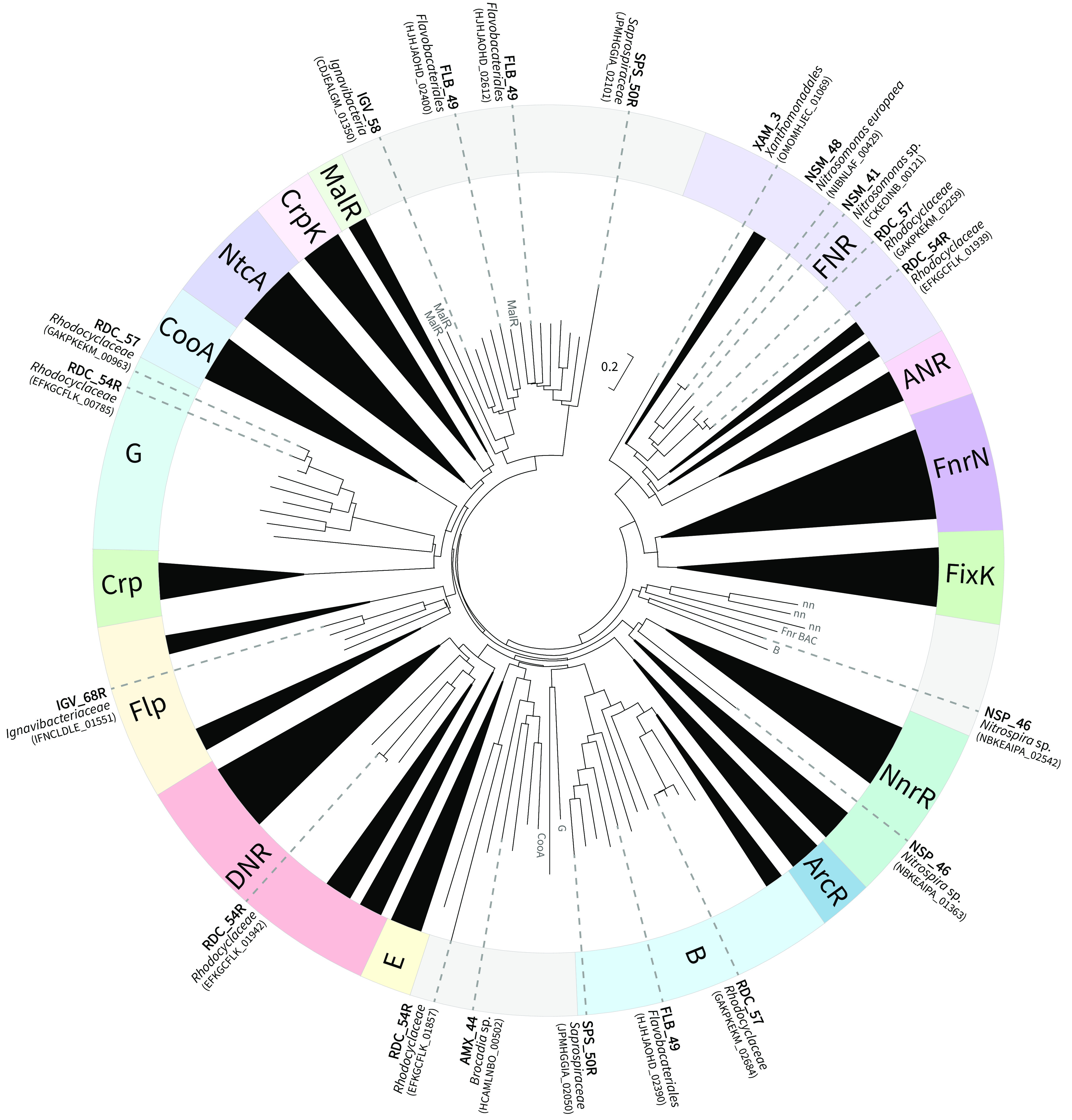
Phylogenetic tree generated from the alignment of transcription factors (TFs) from the CRP/FNR family along with TF homologs found in 11 of the 16 most active MAGs. TF names in outer ring are based on the phylogenetic analysis of Körner et al. (11). Subtrees that lacked an identifiable clustering of TFs contain gray shading in the outer ring and do not have a label.

The observed lack of correlation between relative mRNA and DNA abundances led us to hypothesize that TFs play an important role in the activity of the microbiome. To evaluate this hypothesis, we focused on members of the CRP/FNR family of TFs because they are both widespread phylogenetically and often respond to the stimuli cycled in the bioreactor, specifically oxygen, nitrogen, and carbon availability. Homologs of the CRP/FNR family of TFs were identified in 29 of the 43 high-quality MAGs (Fig. S1), including in 11 of the 16 most active MAGs (Fig. 4). The environmental stimuli these TFs respond to were partially inferred from comparison to the phylogenetic analysis of Körner et al. (11) (Fig. 4; Fig. S1). Further inference was based on conservation of important amino acid residues that have been identified as essential for function of homologs of these TFs in pure cultures (Table S4). Below, we will focus on the homologs found in the most active MAGs (Fig. 4); a more comprehensive phylogenetic analysis for all 29 MAGs with identified CRP/FNR homologs can be found in Fig. S1.

10.1128/mSystems.00906-21.1 FIG S1 Full phylogenetic tree clustering CRP/FNR homologs found in all members of the microbiome with those from Körner et al. (11). Download FIG S1, EPS file, 0.6 MB. Copyright © 2021 Beach et al. 2021 Beach et al. https://creativecommons.org/licenses/by/4.0/ This content is distributed under the terms of the Creative Commons Attribution 4.0 International license.

10.1128/mSystems.00906-21.6 TABLE S4 Summary of identified CRP/FNR homologs in the top 16 MAGs based on relative RNA abundance. Download Table S4, XLSX file, 0.02 MB. Copyright © 2021 Beach et al. 2021 Beach et al. https://creativecommons.org/licenses/by/4.0/ This content is distributed under the terms of the Creative Commons Attribution 4.0 International license.

FNR is a global TF associated with bacterial response to oxygen limitation (25, 26). We found that homologs from two *Nitrosomonas* MAGs (NSM_48 and NSM_41) and two *Rhodocyclaceae* MAGs (RDC_57 and RDC_54R) clustered within known FNR TFs (Fig. 4), including an FNR protein from N. europaea ATCC 25978 (Fig. S1). Examination of the protein sequences of these FNR homologs revealed the presence of at least four cysteine residues (Table S4), which is a critical characteristic that has been associated with coordination of a 4Fe-4S cluster that allows them to sense oxygen (27). These results provide evidence that the *Nitrosomonas* and *Rhodocyclaceae* members of this PNA microbiome could use FNR to sense O_2_ as part of a transcriptional response to the alternating DO conditions created by the air on/air off cycles in the bioreactor. One MAG (XAM_3) contained a predicted CRP/FNR homolog that also clustered near the FNR proteins in the phylogenetic tree (Fig. 4). However, because this homolog lacked the conservation of the four cysteine residues characteristic of FNR proteins, we did not pursue additional examination of this CRP/FNR homolog.

The DNR and NnrR TFs have been associated with bacterial sensing of nitric oxide (NO), an intermediate in nitrogen cycling metabolism (28, 29). Only one CRP/FNR homolog found in one of the *Rhodocyclaceae* MAGs (RDC_54R) clustered near known DNR TFs (Fig. 4). Comparing the protein sequence of this homolog to that of the DNR protein from Pseudomonas aeruginosa PAO1 revealed conservation of a histidine residue at position 139 (Table S4), which has been shown to be important in coordination of the heme cofactor required to sense NO (30), suggesting this DNR homolog could respond to changes in NO. This further indicates that *Rhodocyclaceae* (RDC_54R) contains both an FNR homolog and a DNR homolog, raising the possibility that it can respond to both oxygen and NO concentrations. We also found that a CRP/FNR homolog in the *Nitrospira* MAG (NSP_46) clustered with proteins that have been identified as the TF NnrR (Fig. 4) (31, 32). Comparison to the well-studied NnrR protein from Rhodobacter sphaeroides shows conservation of the tyrosine residue at position 93 (Table S4), which has been shown to be important for function during *in vivo* regulatory activity (32). These data suggest that *Nitrospira* (NSP_46) uses NnrR to sense and respond to changes in nitrogen concentration during the cyclic operation of the bioreactor. Another CRP/FNR TF from NSP_46 clustered near FNR_BAC_. FNR_BAC_ is distinct from FNR from Escherichia coli in that it is activated by a two-component regulatory system (ResDE) and has the sensor domain at the C terminus (33) (Fig. 4; Table S4). Since we did not find homologs of ResDE in NSP_46 and the TF from NSP_46 does not have conserved cysteine residues in the C terminus, we did not include it in further analysis.

MalR is a member of the CRP/FNR TF family that responds to sugars and other carbon sources (34). Several CRP/FNR homologs in the microbiome clustered near other TFs annotated as MalR but that are outside the main MalR cluster (Fig. 4). These were homologs in *Ignavibacteria* IGV_58, *Flavobacteriales* FLB_49, and *Saprospiraceae* SPS_50R. Little is known about how MalR responds to environmental or other signals, but there is significant amino acid sequence identity between a predicted effector binding domain in MalR from Bacteroides thetaiotaomicron and the homologs from MAGs clustered near other MalR proteins (Table S4) (34, 35). Thus, we hypothesize that these proteins are likely MalR homologs and that these MAGs may respond to changes in the availability of carbon sources, albeit in an indeterminate manner. The *Ignavibacteria* IGV_68R has a CRP/FNR homolog that does not cluster near MalR-related homologs, but within the Flp cluster (Fig. 4). Flp has been shown to coordinate a transcriptional response to oxidative stress in *Lactobacillus*, but in Clostridium perfringens, it has been proposed to play a role in a transcriptional response to carbohydrates like MalR (11). Indeed, this Flp homolog had significant amino acid sequence conservation in the predicted effector binding domain in B. thetaiotaomicron MalR and to the MalR-related TF in IGV_58 (Table S4), suggesting that this TF may also have a function in recognition of carbon source availability.

The CRP/FNR TF homolog in the anammox Brocadia MAG (AMX_44) did not cluster within a group of previously studied TFs (Fig. 4; Fig. S1). The nearest annotated TF was identified previously as CooA from Treponema pallidum (Fig. S1). However, previous analysis of CooA in Rhodospirillum rubrum (36), along with annotations for homologs of this TF (e.g., using the KEGG database [37]), suggests this TF may be a CRP homolog. Using a protein BLAST comparison, we found substantially more conservation between CRP from E. coli (38) and both the TF from T. pallidum and the TF from AMX_44 than conservation with CooA, leading us to conclude this TF is likely a CRP homolog (Table S4). CRP in other organisms is known to respond to changes in carbohydrates, such as glucose, to regulate energy metabolism (39, 40). However, its presence in the autotrophic Brocadia MAG (AMX_44), and its phylogenetic distance from the main CRP cluster (Fig. 4), makes it difficult to formulate a hypothesis about function based solely on comparison of protein sequences.

### Predicting the microbiome’s meta-regulon.

**FIG 5:**
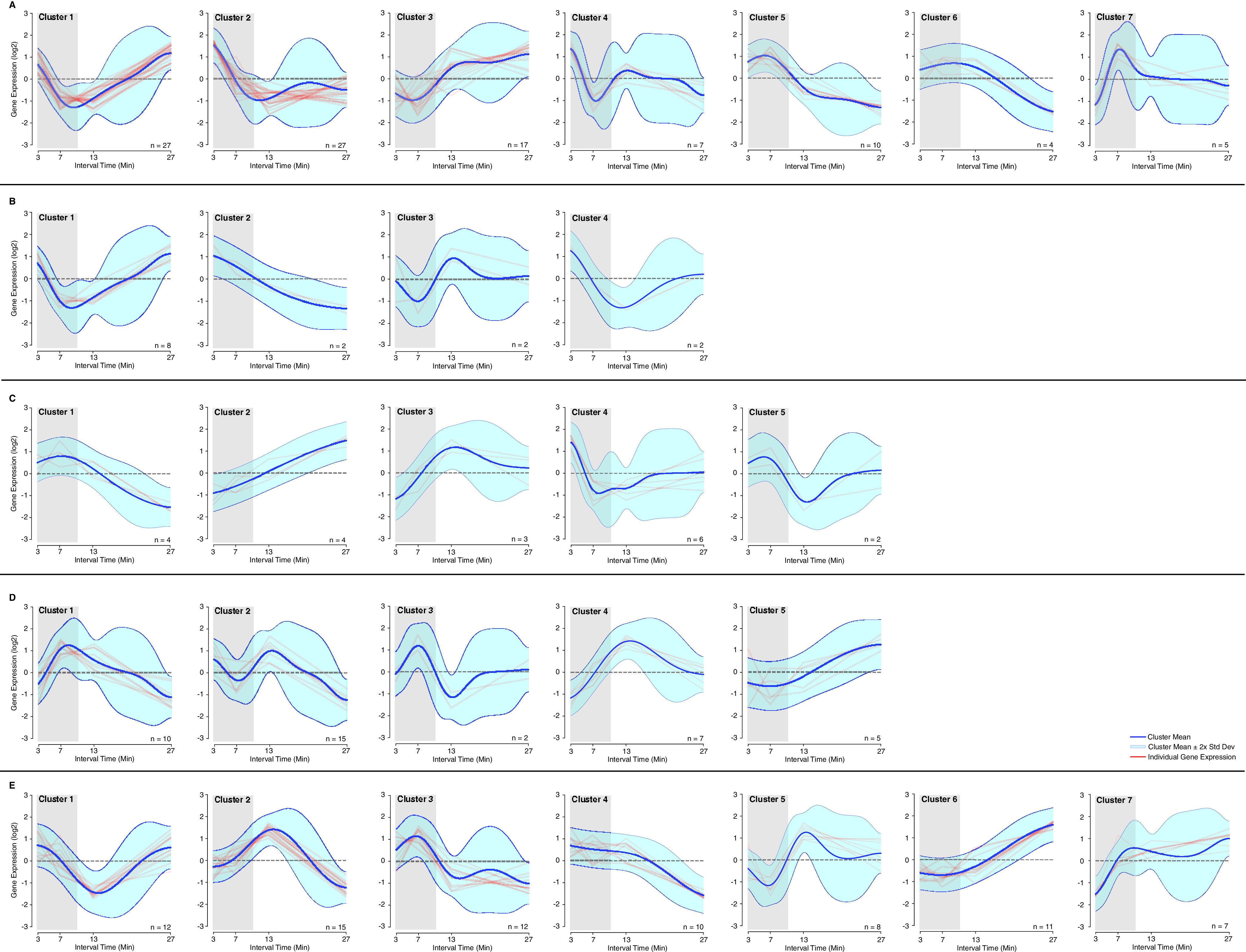
Clustering of time-dependent changes in expression levels in genes that contain upstream CRP/FNR homolog binding sites. The figure includes data from the 11 most active MAGs predicted to have CRP/FNR family TFs. (A) FNR; (B) DNR; (C) NnrR; (D) MalR; (E) CRP. Dark blue line indicates expression mean for each cluster, light blue indicates 2× standard deviation, and red lines indicate individual gene expression patterns. Time points within the shaded region correspond to samples taken during aerated periods, outside the shaded region correspond to unaerated periods. Information on genes within each cluster is found in Table S6.

In addition to identifying CRP/FNR homologs in the most active MAGs and inferring their function based on amino acid sequence comparisons and conservation of residues that have been found essential for activity in well-studied organisms, we sought further evidence of function using the metatranscriptomic data set (Table S3) to evaluate how genes predicted to be regulated by the TFs responded to the cyclic low-DO/anoxic conditions created in the PNA bioreactor. To do this, we generated position weight matrices (PWMs) for CRP/FNR family TFs (Table S5) using published information from either within a single organism or phylogenetic analysis (Fig. S2) (26, 29, 40–46). These PWMs matched previously identified PWMs for these TF homologs (47). These PWMs were used to predict binding sites upstream of genes within each of the most active MAGs for which a CRP/FNR homolog was identified. Next, the gene expression data, obtained during air off/air on cycles of the PNA bioreactor, was used to identify expression patterns (Fig. 5) and categorize the annotated functions of these genes (Table S6).

10.1128/mSystems.00906-21.2 FIG S2 Logos of the PWMs used for each TF in this analysis. Download FIG S2, EPS file, 2.2 MB. Copyright © 2021 Beach et al. 2021 Beach et al. https://creativecommons.org/licenses/by/4.0/ This content is distributed under the terms of the Creative Commons Attribution 4.0 International license.

10.1128/mSystems.00906-21.7 TABLE S5 PWMs and sequences used to construct the PWMs for each TF using in this analysis. Download Table S5, XLSX file, 0.03 MB. Copyright © 2021 Beach et al. 2021 Beach et al. https://creativecommons.org/licenses/by/4.0/ This content is distributed under the terms of the Creative Commons Attribution 4.0 International license.

10.1128/mSystems.00906-21.8 TABLE S6 Metatranscriptomic clustering results. Download Table S6, XLSX file, 0.04 MB. Copyright © 2021 Beach et al. 2021 Beach et al. https://creativecommons.org/licenses/by/4.0/ This content is distributed under the terms of the Creative Commons Attribution 4.0 International license.

This analysis predicts the TF regulons within each community member and allows us to infer action and environmental stimuli for individual MAGs within the PNA bioreactor. As described above, FNR homologs were predicted in *Rhodocyclaceae* (RDC_54 and RDC_57) and *Nitrosomonas* (NSM_48 and NSM_41) MAGs. Using a PWM from binding sites in E. coli (26), FNR binding sites were identified upstream of 65 genes in the *Nitrosomonas* MAGs and 30 genes in *Rhodocyclaceae* MAGs (Table S6). While a large fraction of these genes encoded hypothetical proteins and could not be analyzed further, we were able to infer how FNR controls the expression of several genes with annotated function in the *Nitrosomonas* and *Rhodocyclaceae* MAGs.

In the *Rhodocyclaceae* MAGs, genes predicted to be controlled by FNR and with increased expression in the anoxic conditions (clusters 1 and 3 in Fig. 5A) include those encoding the following proteins: nitrite reductase (gene identification in Table S6, EFKGCFLIK_01293, EFKGCFLIK_01298, GAKPKEKM_00701, and GAKPKEKM_00706), nitrous oxide reductase (EFKGCFLK_00100), and high-efficiency Cbb3 cytochrome oxidase (EFKGCFLIK_01956 and GAKPKEKM_02276). One annotated gene with increased expression under aerobic conditions (cluster 2 in Fig. 5A) encodes an acetyl-coenzyme A synthetase (EFKGCFLK_00226), suggesting increased tricarboxylic acid (TCA) cycle activity in the presence of oxygen.

In the *Nitrosomonas* MAGs, genes predicted to be controlled by FNR and with increased expression in the anoxic conditions (cluster 3 in Fig. 5A) include those encoding the following proteins: an anaerobic TF in the CRP family ANR (FCKEOINB_00121), which was not detected in our initial analyses; NADH-quinone oxidoreductase (FCKEOINB_00346) involved in respiration; and a chaperone involved in photosystem 1 formation in other organisms (JNIBNLAF_02096). Among the FNR-controlled genes in *Nitrosomonas* with higher expression under aerobic conditions (cluster 6 in Fig. 5A) was a cytochrome *c*_551_ peroxidase, which is often involved in responses to oxidative stress (FCKEOINB_01367). Interestingly, when comparing the FNR-controlled gene expression patterns from the *Nitrosomonas* and *Rhodocyclaceae* MAGs, most genes with higher expression in the anoxic conditions came from the *Nitrosomonas* MAGs (clusters 4, 5, 6, and 7 in Fig. 5A; Table S6). Among them, there were genes whose products are involved in amino acid biosynthesis (JNIBNLAF_00839, JNIBNLAF_00216) (clusters 4 and 5 in Fig. 5A). Taken together, these data suggest that *Rhodocyclaceae* and *Nitrosomonas* in the microbiome utilize FNR homologs to regulate different sets of genes in response to O_2_ changes.

In the *Rhodocyclaceae* RDC_54R MAG, we also identified a DNR homolog which is predicted to be responsive to NO concentration. Overall, 14 genes were predicted to be regulated by this TF (Table S6) using a PWM constructed from binding sites in both P. aeruginosa and across proteobacteria (44, 48). The algorithm used separated gene expression patterns into 4 different clusters with the majority of the genes (8 out of 14) included in a single cluster (cluster 1 in Fig. 5B). The clustering results suggest that DNR increases the anoxic expression of genes encoding the following proteins: a nitrite reductase (EFKGCFLK_01293), an Fe-S cluster assembly protein which may need to participate in metalloprotein repair after O_2_ exposure (EFKGCFLK_01945), a high-efficiency Cbb3-type cytochrome *c* oxidase (EFKGCFLK_01956), and an Na^+^ pump that may be required for nutrient uptake (EFKGCFLK_01284) (Table S6). Additionally, several of these genes were predicted to be regulated by both FNR and DNR, which may be due to the high similarity of the TF motifs (Fig. S2) (44), and suggests a multifaceted response system in RDC_54R.

After identifying a NnrR homolog in the *Nitrospira* NSP_46 MAG, we used the binding site analysis from a PWM constructed from sites in R. sphaeroides and enterobacteria (29, 44) that showed close similarity to other NnrR PWMs (47) to identify 19 genes that may be regulated by this TF (Table S6). NnrR shows a more diverse set of expression patterns in response to the aerobic/anoxic shift in the PNA bioreactor (Fig. 5C). In addition, NnrR appears to increase the anoxic expression of another TF (NorR; NBKEAIPA_00806) and regulates expression of genes that encode an anaerobic nitric oxide reductase, a putative oxidoreductase (NBKEAIPA_03429), and a DNA repair protein RecO (NBKEAIPA_01701) (cluster 2 of Fig. 5C; Table S6). NnrR appears to increase the anoxic expression of the gene that encodes a NAD-dependent malic enzyme (NBKEAIPA_01292) (cluster 3 in Fig. 5C; Table S6) predicted to decarboxylate malic acid to pyruvic acid, an enzyme that could recycle central carbon metabolites during periods when oxygen is limiting or not available as the terminal electron acceptor.

Since several of the CRP/FNR homologs identified in the most active MAGs were loosely associated with MalR, we used a PWM of MalR deduced from both Bacteroides thetaiotaomicron and other *Bacteroides* species (43, 46) (Fig. S2) to query two *Ignavibacteria* MAGs (IGV_58 and IGV_68R), a *Flavobacteriales* MAG (FLB_49), and a *Saprospiraceae* MAG (SPS_50R) (Table S6). This analysis revealed 31 genes to be predicted targets for MalR homologs, which formed 5 clusters of gene expression patterns (Fig. 5D). The genes predicted to be controlled by MalR homologs were mostly hypothetical proteins (Table S6). However, in FLB_49, we identified a gene encoding a dicarboxylate transporter (HJHJAOHD_01762) among the genes with an overall decrease in expression during anoxic conditions (cluster 2 in Fig. 5D) and a gene encoding a dihydrolipoyl dehydrogenase (HJHJAOHD_02680) among the genes with a transitory increase in expression (cluster 4 in Fig. 5D), suggesting a possible connection of this transcription response to carbohydrate availability. However, the clustering analysis also suggests MalR leads to aerobic/anoxic expression changes of genes whose products are involved in translation and protein folding (CDJEALGM_00124, JPMHGGIA_00186, and JPMHGGIA_02733 in cluster 1 of Fig. 5D; CDJEALGM_00251 in cluster 3 of Fig. 5D), purine biosynthesis (CDJEALGM_01756 and HJHJAOHD_00568 in cluster 1 of Fig. 5D), cell division (IFNCLDLE_02350 in cluster 1 of Fig. 5D), and vitamin B_12_ transport (JHJHAOHD_01619 in cluster 1 of Fig. 5D) (Table S6). Our data predict that MalR may also play a role in increasing the expression of genes during anoxic conditions (cluster 5 of Fig. 5D) whose products are involved in NADP+ biosynthesis (IFNCLDLE_02041) and a protein with homology to Ycf48-like proteins (IFNCLDLE_02041), which are typically involved in photosystem II formation in photosynthetic bacteria. These results suggest that MalR may play an expanded role in the *Ignavibacteria*, *Flavobacteriales*, and *Saprospiraceae* MAGs and that MalR may be responding to stimuli other than O_2_ in the changing anoxic/aerobic conditions to regulate the expression of genes that encode proteins with diverse functions.

The CRP/FNR homolog identified in the Brocadia MAG (AMX_44) showed a high percentage of amino acid identity with CRP from E. coli (Table S4), suggesting this TF senses and responds to organic carbon sources. Using a PWM for the CRP of E. coli (Fig. S2) (49), we identified and clustered 72 genes apparently controlled by the CRP/FNR homolog in AMX_44 (Fig. 5E; Table S6). The genes predicted to be regulated by the CRP homolog formed 7 different clusters of gene expression patterns (Fig. 5E). Genes with increased expression in the anoxic conditions encode proteins required for lactate utilization (HCAMLNBO_00511), processing sugar alcohols (HCAMLNBO_01545), and trehalose biosynthesis (HCAMLNBO_02797) (clusters 5 and 6 in Fig. 5E; Table S6). Alternatively, genes encoding proteins involved in cell survival during nutrient stress (HCAMLNBO_02721), high osmolarity (HCAMLNBO_02796), the housekeeping sigma factor (HCAMLNBO_00174), coenzyme A biosynthesis (HCAMLNBO_00662 and HCAMLNBO_02270), and 2-oxoglutarate oxidoreductase (HCAMLNBO_00236) were expressed at higher levels during aerobic conditions in the PNA bioreactor (clusters 2, 3, and 4 in Fig. 5E; Table S6).

## DISCUSSION

Here, we have described an anammox microbial community from a PNA bioreactor operating with cycling anoxic/aerobic (low-DO) conditions. Microbial community members were identified using metagenomic analysis, and the connections between relative abundances based on DNA and mRNA levels were analyzed. Similar differences in relative abundance of organism and transcript based on DNA and mRNA analysis of the PNA bioreactor to those found in this study have been described in other microbiomes (50, 51), including deammonification communities such as the one studied here (6). This observation is especially interesting in the PNA microbiome since nitrogen removal could have easily been attributed to conventional nitrification and denitrification due to the presence of COD in the influent (Table 1) and because the metagenomic analysis predicted that anammox microbes were not abundant (Fig. 3).

This disconnect between relative DNA abundance and gene expression levels prompted us to develop a CRP/FNR family meta-regulon of the PNA bioreactor community since the CRP/FNR family of TFs is widely conserved across bacteria and because individual members of this group of proteins are known to control transcriptional responses to the presence of oxygen, alternative electron acceptors, and carbon sources (11). The identified predicted meta-regulon sheds new light on the biology of understudied organisms within the community as well as how these organisms interact with environmental stimuli and with other organisms in this microbiome. Below, we will discuss how these analyses contribute to our understanding of this microbial community and how they could be applied to better describe other microbiomes and their microbial interactions.

### FNR regulates *Nitrosomonas* activity under anoxic conditions.

The microbial functions that are essential for the establishment of PNA in a bioreactor are aerobic oxidation of ammonium to nitrite and anaerobic deammonification of ammonium and nitrite to nitrogen gas. Other microbial activities in a PNA microbiome are complementary to the main function of the bioreactor and will depend on the availability of organic carbon sources and the parameters of the aerobic/anoxic cycles. N. europaea was identified as the bacterium performing aerobic ammonia oxidation, represented by the NSM_48 and NSM_41 MAGs, whereas Brocadia (AMX_44) was identified as performing deammonification. An FNR TF was identified in the N. europaea MAGs, indicating that this organism directly senses and responds to O_2_ availability. The predicted FNR regulon did not include any of the core enzymes for ammonia oxidation (e.g., ammonia monooxygenase or hydroxylamine dehydrogenase) or CO_2_ fixation (e.g., rubisco), which are core pathways for which different transcript abundances have been detected under ammonia-limited or oxygen-limited conditions (52). Our prediction that core ammonia oxidation genes are not regulated by FNR is consistent with prior research with N. europaea pure cultures that has shown transcription of these genes to be regulated by ammonia concentrations (53). Likewise, CO_2_ fixation genes in N. europaea have been shown to be regulated by the master carbon fixation regulator *cbbR* (54), which responds to CO_2_ levels, although it may also have a complex regulatory network (55). A gene predicted to be under the control of FNR and with higher transcript abundance under aerobic conditions encodes cytochrome *c*_551_ peroxidase, indicating N. europaea mounts a transcriptional response to the presence of reactive oxygen species, even under the low-DO conditions used in the bioreactor.

**FIG 6:**
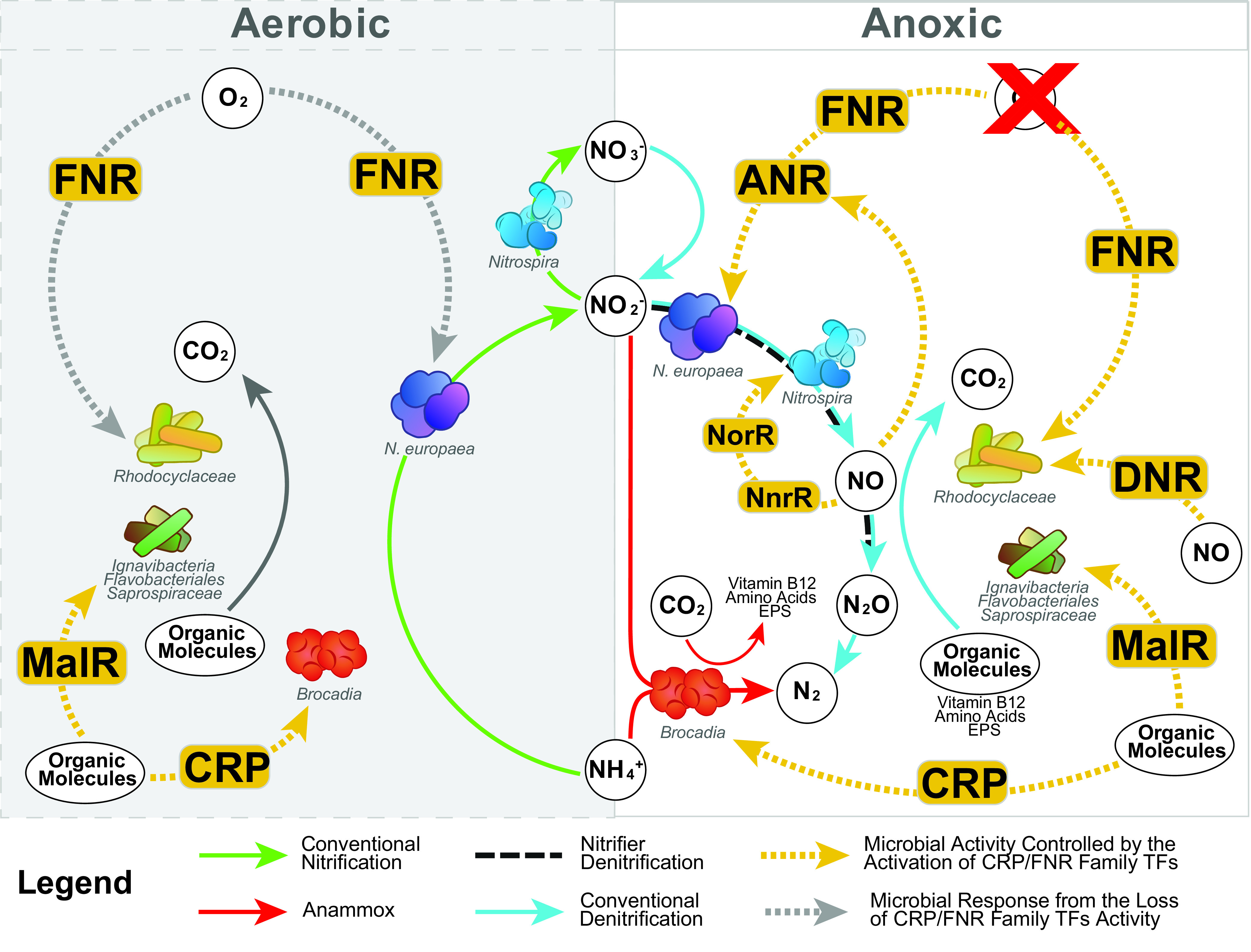
Summary of CRP/FNR TF family meta-regulon in the PNA bioreactor. TF homologs are represented with yellow rectangles. Yellow or gray dotted lines indicate the environmental stimulus sensed by each TF and which organisms the TFs were identified within. Organisms are labeled, along with key chemical reactions taking place within the PNA bioreactor. Green arrows indicate conventional nitrification, red arrows indicate anammox activity, black dashed lines indicate nitrifier denitrification, and light blue arrows indicate conventional denitrification. The presence (left) or absence (right) of O_2_ is indicated.

Pure cultures of N. europaea have also been shown to mount a transcriptional response to changes from aerobic to anoxic conditions, which include increased expression of genes that encode nitrite reductase and reduction of nitrite to NO (24). Our analysis did not predict a direct control of nitrifier denitrification genes by FNR, but the anaerobic transcription factor ANR was predicted to be FNR regulated. Anaerobic regulator of arginine deiminase and nitrate reductase (ANR) (56) is another TF in the CRP/FNR family that has been studied in P. aeruginosa and is shown to regulate nitrite reductase activity (57). Thus, it is possible that a regulatory cascade exists in which FNR activates transcription of ANR, and ANR regulates the expression of the N. europaea MAGs nitrifier denitrification genes in a PNA bioreactor (Fig. 6), similar to what has been observed in E. coli (26).

### FNR and DNR regulate *Rhodocyclaceae* denitrification.

FNR was also found in the abundant *Rhodocyclaceae* MAGs (RDC_57 and RDC_54R) in the PNA bioreactor for which the predicted FNR regulon included genes that encode nitrite reductase and nitrous oxide reductase. The expression of these genes was found to be higher under anoxic conditions, indicating these MAGs are responding to O_2_ levels and carrying out a conventional denitrification function in the PNA bioreactor (Fig. 6). Transcripts for the *cbb3* gene, a cytochrome oxidase enzyme which typically has a high affinity for oxygen (58), were also found to be higher under anoxic conditions and predicted to be under the control of FNR. Although anoxic upregulation of a cytochrome used in aerobic respiration would seem counterintuitive, the observed coregulation of cbb3 with nitrite reductase and nitrous oxide reductase expression is consistent with observations for another *Rhodocyclaceae* in an unrelated microbiome that was also experiencing cyclic anoxic/low-DO conditions (59). Therefore, it is plausible that *Rhodocyclaceae* are adapted to environments in which cycles of anoxic and aerobic conditions persist and that high expression of the high-affinity cbb3 cytochrome is linked to the ability of these organisms to scavenge O_2_ under O_2_-limiting conditions. These observations also predict that the abundant *Rhodocyclaceae* MAGs are performing conventional aerobic respiration in the presence of oxygen (Fig. 6). Thus, in the context of the PNA bioreactor, *Rhodocyclaceae* may play an important role in COD removal under both aerobic and anoxic conditions. In addition, the increase in anaerobic expression of a gene that encodes an Na^+^ pump raises the possibility that *Rhodocyclaceae* requires Na^+^ for optimal anoxic growth, similar to the Na^+^ requirement for optimal growth in some marine and halophilic bacteria (60).

Some TFs of the CRP/FNR family have a high degree of amino acid sequence similarity in their C-terminal DNA binding domains and recognize similar sequence motifs, although their regulons may be different (57). In such cases, it is difficult to make regulon predictions with only computational tools, and genetic or biochemical experiments are needed to obtain experimental evidence to determine the individual regulons. Such is the case for the abundant RDC_54R MAG, for which DNR and FNR TFs were identified, and the PWMs used to identify regulated genes by both proteins were very similar (Fig. S2). Although the predicted regulons for DNR and FNR shared common genes in the RDC-54R MAG, the prediction that O_2_ controls expression of denitrification genes in this organism is robust, regardless of which TF is controlling specific genes.

### NnrR regulates nitrifier denitrification activity in *Nitrospira*.

We identified an NnrR homolog in a *Nitrospira* NSP_46 MAG that was a canonical NOB that clustered with ‘*Ca*. Nitrospira defluvii’ and with NOB strains detected in other systems, including another PNA bioreactor (19) and a bioreactor operated under alternating anoxic/low-DO conditions (61) (Fig. 2). NnrR is a CRP/FNR TF that is found in denitrifying organisms and is predicted to activate transcription of genes that encode respiratory nitrite reductase and nitric oxide reductase in response to NO accumulation in organisms such as R. sphaeroides, where it is hypothesized to serve as an NO detoxification mechanism (62). Thus, the detection of an NnrR TF predicts that *Nitrospira* responds to NO concentrations and activates genes that encode reductive pathways to prevent NO toxicity (Fig. 6).

Further support for the above hypothesis is our finding that the gene encoding a homolog of NorR is predicted to be under the control of NnrR the abundant *Nitrospira* MAG NSP_46 (Table S6). NorR is a TF that has been found to regulate various enzymes that use NO as a substrate in other organisms and, hence, has been described as a dedicated NO sensor (62). Although NSP_46 and related *Nitrospira* MAGs from other studies (19, 61) encode a copper-containing dissimilatory nitrite reductase (*nirK*), which catalyzes the reduction of nitrite to nitric oxide, an NO reductase gene has not been identified in *Nitrospira* (61). Thus, although the evidence suggests *Nitrospira* in a PNA bioreactor responds to changes in O_2_ and NO, there is not sufficient information to hypothesize how *Nitrospira* controls potential NO toxicity during the anoxic periods in the bioreactor when NO concentrations are expected to increase. Interestingly, NnrR appears to decrease the anaerobic expression of a gene encoding a peptidoglycan transglycosylase, which may be involved in the cell cycle (63), suggesting that NnrR may indirectly decrease cell division in *Nitrospira* during anoxic conditions.

### MalR regulates the activity of *Ignavibacteria* and other organisms in the PNA microbiome.

From the perspective of CRP/FNR homologs, the IGV_58, FLB_49, and SPS_50R MAGs in the PNA bioreactor all appeared to encode TFs that could be related to MalR (11, 34, 43, 46, 64), so we hypothesize that these organisms mount a transcriptional response to the cyclic anoxic/aerobic conditions that depends on changing concentrations of organic carbon molecules. Besides a large number of hypothetical proteins, the predicted MalR-related regulon in these organisms contained genes that coded for functions related to carbohydrate metabolism (dicarboxylate transporter and dihydolipoyl dehydrogenase) and proteins involved in vitamin B_12_ transport and purine biosynthesis. These observations support a proposed synergistic relationship between an *Ignavibacteria* (UTCHB1 in Fig. 2) and anammox (UTAMX1 in Fig. 2) in an anaerobic deammonification bioreactor (6) in which the *Ignavibacteria* reduced nitrate to nitrite, providing a needed substrate for the anammox bacteria, whereas the exopolymeric substances (EPS) generated by anammox provided an organic carbon source needed to support growth of the *Ignavibacteria*. In addition, the previous analysis indicated that *Ignavibacteria* did not have complete pathways for vitamin and amino acid biosynthesis, and hypothesized that vitamin B_12_ generated by anammox could be assimilated by the *Ignavibacteria* (6). Although we did not find any evidence of denitrification regulation by CRP/FNR family TFs in these organisms, our analysis of this PNA bioreactor are consistent with the hypothesis that organic metabolite exchanges between autotrophic and heterotrophic organisms in deammonification systems may contribute to shaping microbial community assemblies in these microbiomes (6).

### Anammox mounts a transcriptional response to aeration cycles in the PNA microbiome.

Brocadia AMX_44, the anammox organism detected in the PNA microbiome, was the only abundant MAG with a predicted CRP homolog that may respond to changes in carbon source via the abundance of cyclic AMP (cAMP) (65). The predicted presence of a CRP in Brocadia AMX_44 is interesting because growth of this autotrophic organism has been shown to be potentially inhibited by organic carbon (66–68). Recent work, however, suggests that anammox organisms could utilize formate as a carbon source (69). In support of the latter notion, we observed a predicted CRP-dependent increase in anoxic expression of genes encoding proteins involved in coenzyme A biosynthesis and 2-oxoglutarate oxidoreductase, which may play a role in formate or CO_2_ utilization in the bifurcated TCA cycle known to be present in many anammox bacteria (70). However, the presence of a CRP regulon suggests a previously unreported role for a transcription response in the anammox organism that may not be directly connected to organic carbon utilization. These results suggest a previously unknown and potentially important response to aerobic/anoxic cycling in Brocadia for which additional research is needed to shed light on the role of the predicted CRP-related TF.

### Additional research to expand our knowledge of the PNA bioreactor meta-regulon.

This study provides a first glimpse of the coordinated response of members of a PNA microbiome to changes in environmental stimuli brought about by establishing aeration cycles. Our hypothesis that CRP/FNR family TFs would play a central role in the transcriptional response of community members was confirmed by finding TFs of this family in 11 of the most active 16 MAGs in this PNA microbiome. Although the remaining 5 most active MAGs (ANR_43, BRB_32R, ANR_55, STB_2, and ANR_56R) (Table 2) did not have an identifiable CRP/FNR family TF, gene annotations reveal the presence of other TFs that may be responsive to expected environmental changes in the cyclic aeration environment. For instance, homologs to the nitrate/nitrite-responsive TF NarL (71) are present in all five MAGs, the nitric oxide-responsive TF NsrR (25) is found in BRB_32R and STB_2, the iron-responsive TF Fur (72) is present in ANR_43 and BRB_32R, and the Fe-S cluster- and oxidative stress-responsive TF IscR (73, 74) is found in BRB_32R, ANR_55, and STB_2. This suggests that these other TFs may play an important role either alone or through coregulation with other TFs in these (and likely other) members of this PNA microbial community. However, it is unclear how these additional TFs may contribute to the microbiome response to the aerobic/anoxic environmental changes or how they may interact with other TFs, including CRP/FNR family TFs, to regulate gene expression. Further, it is also likely that there are multiple levels of regulation, wherein a cascade of TF action leads to widespread gene expression regulation. Such a mechanism has been observed previously (26), and additional research could determine how similar mechanisms regulate microbial community members.

The methodology we used, which relied on using PWMs obtained from well-studied bacterial TFs to search for DNA binding sites upstream of genes in MAGs, predicted the regulon of different CRP/FNR family TFs and, in many cases, supported hypotheses for the role of PNA community members that were inferred from either the physiology of the individual community members, the presence/absence of specific genes, or transcript abundance measurements (73, 74). Connecting the presence of an environmental factor like oxygen to the global transcriptional response of specific genes is an important step in unraveling how a microbiome is wired to respond, in a concerted manner, to natural or imposed stimuli. Thus, predicting the meta-regulon of microbiomes is a critical part of future work to develop strategies to engineer the composition and function of microbiomes for practical applications (75).

With analyses of pure cultures, *in silico* predictions of the regulon of a specific TF could be tested experimentally by genetic and other approaches. However, it is often difficult or impossible to study the transcriptional networks of a microbiome since most of the major organisms are usually not culturable. Further research is needed to develop strategies that allow experimental verification of DNA binding sites of TFs in specific community members. Thus, adapting high-throughput approaches such as chromatin immunoprecipitation (ChIP) combined with DNA sequencing (ChIP-seq) (76) or *in vitro* DNA affinity purification sequencing (DAP-seq) (77) to study the presence and function of transcriptional networks in microbiomes would enable testing and refinement of our meta-regulon predictions.

Finally, the strategies and methodologies used in this research could be used to begin elucidating transcriptional regulatory networks in other microbiomes. From the perspective of wastewater treatment, knowing how key members of the communities are wired to respond to cyclic environments could help identify optimal conditions for creating the desired aeration cycles and simplify the trial-and-error basis on which current practices and new treatment processes are developed and optimized.

## MATERIALS AND METHODS

### PNA bioreactor description and operation.

A 3-liter lab-scale bioreactor was originally inoculated with biomass obtained from the full-scale York River Treatment Plant (Hampton Roads Sewerage District, Seaford, VA), which uses a PNA deammonification process (DEMON) to treat reject water from the solids dewatering facility (78). The lab-scale bioreactor was set up to treat reject water from a full-scale struvite recovery process at the Nine Springs Wastewater Treatment Plant (Madison, WI). Each week, a 20-liter sample of reject water was collected, maintained at 4°C, and fed to the bioreactor throughout the week.

The bioreactor was a sequencing batch reactor operated with three 8-h cycles per day. Each cycle consisted of a 4-h fill period, where approximately 1 liter of feed was pumped into the bioreactor, followed by a 3.5-h postfill period. Subsequently, the biomass settled for 3 min before 1 liter of supernatant was decanted over a 12-min period. Then, the reactor remained idle for 15 min before beginning the next 8-h cycle. With one-third of the reactor filled and decanted at each cycle, the hydraulic retention time (HRT) was 24 h. The solids retention time (SRT) was not controlled, with biomass leaving the reactor only during sampling or with the decanted supernatant. Intermittent aeration (10 min on, 20 min off) was maintained during the 7.5-h fill/postfill periods. During aeration, air was supplied with an aquarium pump at a flow rate between 0.1 and 0.15 liter/min. During the 10-min aerated period, microaerobic conditions were maintained using a LabVIEW control program (National Instruments, Austin, TX) to turn off air delivery if DO was above 0.2 mg/liter and turn it on when DO was below 0.2 mg/liter. The bioreactor was heated to keep the water temperature between 30 and 35°C. The pH was uncontrolled but remained at an average of 7.5 ± 0.3. A magnetic stir bar and stir plate were used to mix the culture at 250 to 300 rpm in the 7.5-h fill/postfill periods.

To evaluate reactor efficiency, tests of total chemical oxygen demand (tCOD), soluble chemical oxygen demand (sCOD), ammonium-N (NH_4_^+^-N plus NH_3_-N), TSS, and VSS were conducted following Standard Methods for the Examination of Water and Wastewater (79). Nitrite (NO_2_-N) and nitrate (NO_3_-N) were measured with high-performance liquid chromatography (HPLC) using a RestekUltra Aqueous C_18_ column (Restek Corporation, Bellefonte, PA) and detection by UV at 214 nm in a Shimadzu HPLC system (Shimadzu Scientific Instruments, Columbia, MD).

### Metagenomic DNA and mRNA sequencing.

We collected four 6-ml aliquots of biomass at day 77, day 231, day 350, and day 454 of reactor operation, each of which were centrifuged, decanted, and stored at −80°C until DNA extraction. Twenty-four samples were also collected at 454 days, each of which was immediately centrifuged, decanted, flash-frozen in liquid nitrogen, and stored at −80°C prior to RNA extraction.

For DNA extraction, we used a published phenol-chloroform method (80) with some modifications. Cells were lysed by incubation in a mixture of lysis solution (1.5 M sodium chloride, 100 mM Tris, 100 mM EDTA, 75 mM sodium phosphate, 1% cetyltrimethylammonium bromide [CTAB], lysozyme [100 mg/ml; Thermo Fisher Scientific, MA, USA], 20% sodium dodecyl sulfate [SDS; Bio-Rad, Hercules, CA], and proteinase K [New England Biolabs, MA, USA]). A 24:24:1 solution of phenol, chloroform, and isoamyl alcohol was added to each sample, followed by bead beating, repeated centrifugation and aqueous-phase separation steps, incubation, washing, drying, and resuspension. DNA quantity, purity, and quality were assessed with a Qubit 4 fluorometer (Thermo Fisher Scientific, MA, USA), a NanoDrop 2000 spectrophotometer (Thermo Fisher Scientific, MA, USA), and gel electrophoresis.

RNA extraction was performed as described elsewhere (80). RNA quantity, purity, and quality were assessed with a Qubit 4 fluorometer (Thermo Fisher Scientific), a NanoDrop 2000 spectrophotometer (Thermo Fisher Scientific), and gel electrophoresis. RNA samples were submitted to the University of Wisconsin Gene Expression Center for quality control with a Bioanalyzer (Agilent, CA, USA), and rRNA reduction was performed with a RiboZero bacteria rRNA removal kit (Illumina, CA, USA) with a 1-mg RNA input. Strand-specific cDNA libraries were prepared with a TruSeq RNA library preparation kit (Illumina, CA, USA).

Both DNA and cDNA were sequenced with an Illumina HiSeq 2500 platform (Illumina, CA, USA) at the University of Wisconsin Biotechnology Center. For DNA, an average insertion size of 550 bp was used, and 2 × 250-bp reads were generated. For cDNA, 1 × 100-bp reads were generated. Raw DNA and cDNA (mRNA) read data were deposited as a BioProject at National Center for Biotechnology Information (NCBI) (see “Data availability” below).

### Processing of metagenomic data.

DNA samples from the four sampling days were sequenced twice, resulting in 8 DNA sequencing read files. Low-quality DNA sequencing reads were removed using Sickle (81), with a minimum quality score of 20 and a minimum sequence length of 100. Reads from all eight files were then coassembled using SPAdes v3.3.0 (82) in metaSPAdes mode with a range of kmer values (21, 33, 55, 77, 99, and 127) to generate an optimal assembly.

Metagenomic data were binned using Anvi’o v5.5.0, following a modified version of the workflow described by Eren et al. (83) (http://merenlab.org/2016/06/22/anvio-tutorial-v2). Briefly, after simplifying the assembly scaffolds headers with anvi-script-reformat-fasta, the reads that passed quality control criteria were mapped back to the assembly using the bbwrap option of BBMap v38.22 (84), with mapping files converted to BAM file format, sorted, and indexed using SAMtools v1.9 (85). Afterward, a contigs database was generated for each of the four samples included in the coassembly using anvi-gen-contigs-database, which also calls open reading frames using Prodigal v2.6.3 (86). Single-copy bacterial and archaeal genes were identified using HMMER v3.2.1 (87). NCBI Clusters of Orthologous Groups (COGs) of proteins were profiled and included in the contigs databases (88). A profile for each sample was constructed with contigs >2.5 kbp using anvi-profile, which hierarchically clusters contigs based on their tetra-nucleotide frequency profiles. The individual profiles were then merged with anvi-merge, where scaffolds were automatically clustered using a Euclidean distance and Ward linkage algorithm in addition to the CONCOCT algorithm, which uses contig coverage and composition (89). Finally, we manually binned the clusters with the Anvi’o interactive interface by running anvi-interactive. The quality, completeness, and redundancy of each bin were evaluated with anvi-summarize. Initial bins were evaluated for manual refinement using anvi-refine (an added “R” to the end of the bin name denotes additional refinement). Taxonomy for each MAG was determined using the Genome Taxonomy Database (release 05-RS95) (90) with the default settings to identify MAGs with taxonomic classification. MAGs were then further quality controlled using ProDeGe (91) and tetranucleotide frequency comparison to identify and manually remove potential contaminant contigs, all of which were less than 10,000 bp in length. CheckM v1.0.3 (92) was used to determine the completeness, contamination, and heterogeneity of each quality-controlled MAG. Ultimately, we constructed 43 high-quality MAGs with taxonomic classification for further analysis (Table S2 in the supplemental material). We performed a final mapping of all MAGs to calculate the mean coverage and detection and determined DNA-based relative abundance by normalizing the total number of mapped reads for each MAG by the genome size. The metagenomic assembly summary and statistics are presented in Table S2.

### Genome annotation and homology search.

The 43 high-quality MAGs were annotated with Prokka v1.13.3 (93), which calls HMMER v3.2.1 (87) to search sequences against HMM profile databases, BLASTp v2.7.1+ (94, 95), Aragorn v1.2.38 (96) to predict tRNAs, Barrnap v0.9 (97) to predict rRNAs, and Prodigal v2.6.3 (86) for predicting open reading frames (ORFs). Operons for each MAG were predicted using Operon-mapper with default settings (98).

### Metatranscriptomic data processing.

RNA was extracted from triplicate biomass samples collected at 8 time points and processed as described above, resulting in 24 mRNA-sequencing read files. Low-quality mRNA sequencing reads were removed with Sickle using the single end option “se” (81). Read quality was verified with FastQC (99). SortMeRNA was used to remove sequences corresponding to rRNA (100), and the remaining non-rRNA sequences were mapped to the draft genomes using BBMap v35.92 (84) with the minimum sequence identity set to 0.95 and ambiguous mappings randomly assigned. Relative transcript abundance (i.e., gene expression estimates) was calculated for each MAG based on the total number of reads mapped, normalized by the genome size (Table S2). The number of mRNA reads mapping to each ORF within each MAG was calculated with htseq-count v0.6.1 with “-m intersection-strict -s no -a 0 -t CDS -i ID” parameters (101). Gene counts were normalized with reads per kilobase per million (RPKM) (102).

### TF binding motif generation and binding site prediction.

We search for predicted CRP/FNR TF family homologs in all 43 MAGs, and they were identified within 29 MAGs (and 11 of the top 16 MAGs based on expression level) using the gene annotations and searching for FNR, CRP, DNR, NnrR, and MalR descriptors (Table S4). For each TF, a PWM was constructed to represent the TF binding motifs using previously identified binding sites (Fig. S2). The PWMs for FNR and CRP were obtained from studies in E. coli (26, 40–42), and the PWMs for DNR, NnrR, and MalR were constructed from phylogenetic studies to provide enough sequences for a reliable PWM (29, 43–46) (Table S5).

The prediction of TF binding sites was performed as described by Myers et al. (26). Briefly, the sequences 300 bp upstream of each gene within each of the 11 MAGs identified as containing a TF homolog and in the top 16 based on expression level were searched for predicted TF binding sites using the program Patser (103) using a score threshold of 4.5. For each set of TF binding sites predicted with Paster within each MAG, sites were retained as likely binding sites for the TF if their score was greater than two standard deviations of the average of all scores. This resulted in between 49 and 309 predicted binding sites across all the MAGs for the five TF PWMs searched.

### Gene expression time series data clustering.

Clustering analysis was performed using DP_GP_cluster (104) using default parameters. Genes clustered were those downstream of predicted TF binding site within the MAGs based on transcript abundance and genes for which there were values across all sampling time points used. The replicate values for the 3-min, 7-min, 13-min, and 27-min time points were averaged. There was little difference in the clustering results when performed during the fill and no-fill phases, so the replicates at each time point from these two phases were combined.

### Data availability.

MAGs and annotations can be found on the National Center for Biotechnology Information (NCBI) website under BioProject accession number PRJNA559529. DNA sequencing reads are available through the NCBI sequencing read archive (SRA) study accession number SRP235198. RNA sequencing reads are available through the NCBI SRA accession number SAMN14405149.

## References

[1] Metcalf E. 1991. Wastewater engineering: treatment, disposal, reuse, 3rd ed. McGraw-Hill, New York, NY.

[2] Rittmann B, McCarty P. 2001. Environmental biotechnology: principles and applications. McGraw-Hill Book, Co., New York, NY.

[3] Kuenen JG. 2008. Anammox bacteria: from discovery to application. Nat Rev Microbiol 6:320–326. doi:10.1038/nrmicro1857. 18340342

[4] Keene NA, Reusser SR, Scarborough MJ, Grooms AL, Seib M, Santo Domingo J, Noguera DR. 2017. Pilot plant demonstration of stable and efficient high rate biological nutrient removal with low dissolved oxygen conditions. Water Res 121:72–85. doi:10.1016/j.watres.2017.05.029. 28521237 PMC7388030

[5] Camejo PY, Owen BR, Martirano J, Ma J, Kapoor V, Santo Domingo J, McMahon KD, Noguera DR. 2016. *Candidatus* Accumulibacter phosphatis clades enriched under cyclic anaerobic and microaerobic conditions simultaneously use different electron acceptors. Water Res 102:125–137. doi:10.1016/j.watres.2016.06.033. 27340814 PMC7323474

[6] Lawson CE, Wu S, Bhattacharjee AS, Hamilton JJ, McMahon KD, Goel R, Noguera DR. 2017. Metabolic network analysis reveals microbial community interactions in anammox granules. Nat Commun 8:15416. doi:10.1038/ncomms15416. 28561030 PMC5460018

[7] Busby SJW. 2019. Transcription activation in bacteria: ancient and modern. Microbiology (Reading) 165:386–395. doi:10.1099/mic.0.000783. 30775965

[8] Lee DJ, Minchin SD, Busby SJW. 2012. Activating transcription in bacteria. Annu Rev Microbiol 66:125–152. doi:10.1146/annurev-micro-092611-150012. 22726217

[9] Browning DF, Busby SJ. 2004. The regulation of bacterial transcription initiation. Nat Rev Microbiol 2:57–65. doi:10.1038/nrmicro787. 15035009

[10] Kiley PJ, Beinert H. 2003. The role of Fe–S proteins in sensing and regulation in bacteria. Curr Opin Microbiol 6:181–185. doi:10.1016/s1369-5274(03)00039-0. 12732309

[11] Körner H, Sofia HJ, Zumft WG. 2003. Phylogeny of the bacterial superfamily of Crp‐Fnr transcription regulators: exploiting the metabolic spectrum by controlling alternative gene programs. FEMS Microbiol Rev 27:559–592. doi:10.1016/S0168-6445(03)00066-4. 14638413

[12] Fic E, Bonarek P, Gorecki A, Kedracka-Krok S, Mikolajczak J, Polit A, Tworzydlo M, Dziedzicka-Wasylewska M, Wasylewski Z. 2009. cAMP receptor protein from *Escherichia coli* as a model of signal transduction in proteins–a review. J Mol Microbiol Biotechnol 17:1–11. doi:10.1159/000178014. 19033675

[13] Rinaldo S, Giardina G, Brunori M, Cutruzzola F. 2006. N-oxide sensing and denitrification: the DNR transcription factors. Biochem Soc Trans 34:185–187. doi:10.1042/BST0340185. 16417517

[14] Zumft WG. 2002. Nitric oxide signaling and NO dependent transcriptional control in bacterial denitrification by members of the FNR-CRP regulator family. J Mol Microbiol Biotechnol 4:277–286. 11931559

[15] Matsui M, Tomita M, Kanai A. 2013. Comprehensive computational analysis of bacterial CRP/FNR superfamily and its target motifs reveals stepwise evolution of transcriptional networks. Genome Biol Evol 5:267–282. doi:10.1093/gbe/evt004. 23315382 PMC3590769

[16] Spiro S. 1994. The FNR family of transcriptional regulators. Antonie Van Leeuwenhoek 66:23–36. doi:10.1007/BF00871630. 7747934

[17] Roberts GP, Thorsteinsson MV, Kerby RL, Lanzilotta WN, Poulos T. 2001. CooA: a heme-containing regulatory protein that serves as a specific sensor of both carbon monoxide and redox state. Prog Nucleic Acids Res Mol Biol 67:35–63. doi:10.1016/s0079-6603(01)67024-7. 11525385

[18] Darling AE, Jospin G, Lowe E, Matsen F, Bik HM, Eisen J. 2014. PhyloSift: phylogenetic analysis of genomes and metagenomes. PeerJ 2:e243. doi:10.7717/peerj.243. 24482762 PMC3897386

[19] Speth DR, In 't Zandt MH, Guerrero-Cruz S, Dutilh BE, Jetten MS. 2016. Genome-based microbial ecology of anammox granules in a full-scale wastewater treatment system. Nat Commun 7:11172. doi:10.1038/ncomms11172. 27029554 PMC4821891

[20] Bellucci M, Ofiteru ID, Graham DW, Head IM, Curtis TP. 2011. Low-dissolved-oxygen nitrifying systems exploit ammonia-oxidizing bacteria with unusually high yields. Appl Environ Microbiol 77:7787–7796. doi:10.1128/AEM.00330-11. 21926211 PMC3209145

[21] Park HD, Noguera DR. 2007. Characterization of two ammonia-oxidizing bacteria isolated from reactors operated with low dissolved oxygen concentrations. J Appl Microbiol 102:1401–1417. doi:10.1111/j.1365-2672.2006.03176.x. 17448175

[22] Gieseke A, Purkhold U, Wagner M, Amann R, Schramm A. 2001. Community structure and activity dynamics of nitrifying bacteria in a phosphate-removing biofilm. Appl Environ Microbiol 67:1351–1362. doi:10.1128/AEM.67.3.1351-1362.2001. 11229931 PMC92734

[23] Lucker S, Wagner M, Maixner F, Pelletier E, Koch H, Vacherie B, Rattei T, Damste JS, Spieck E, Le Paslier D, Daims H. 2010. A *Nitrospira* metagenome illuminates the physiology and evolution of globally important nitrite-oxidizing bacteria. Proc Natl Acad Sci USA 107:13479–13484. doi:10.1073/pnas.1003860107. 20624973 PMC2922143

[24] Yu R, Chandran K. 2010. Strategies of *Nitrosomonas europaea* 19718 to counter low dissolved oxygen and high nitrite concentrations. BMC Microbiol 10:70. doi:10.1186/1471-2180-10-70. 20202220 PMC2844404

[25] Fleischhacker AS, Kiley PJ. 2011. Iron-containing transcription factors and their roles as sensors. Curr Opin Chem Biol 15:335–341. doi:10.1016/j.cbpa.2011.01.006. 21292540 PMC3074041

[26] Myers KS, Yan H, Ong IM, Chung D, Liang K, Tran F, Keleş S, Landick R, Kiley PJ. 2013. Genome-scale analysis of *Escherichia coli* FNR reveals complex features of transcription factor binding. PLoS Genet 9:e1003565. doi:10.1371/journal.pgen.1003565. 23818864 PMC3688515

[27] Kiley PJ, Beinert H. 1998. Oxygen sensing by the global regulator, FNR: the role of the iron-sulfur cluster. FEMS Microbiology Rev 22:341–352. doi:10.1111/j.1574-6976.1998.tb00375.x. 9990723

[28] Giardina G, Castiglione N, Caruso M, Cutruzzola F, Rinaldo S. 2011. The *Pseudomonas aeruginosa* DNR transcription factor: light and shade of nitric oxide-sensing mechanisms. Biochem Soc Trans 39:294–298. doi:10.1042/BST0390294. 21265791

[29] Hartsock A, Shapleigh JP. 2010. Identification, functional studies, and genomic comparisons of new members of the NnrR regulon in *Rhodobacter sphaeroides*. J Bacteriol 192:903–911. doi:10.1128/JB.01026-09. 19966004 PMC2812982

[30] Rinaldo S, Castiglione N, Giardina G, Caruso M, Arcovito A, Longa SD, D'Angelo P, Cutruzzola F. 2012. Unusual heme binding properties of the dissimilative nitrate respiration regulator, a bacterial nitric oxide sensor. Antioxid Redox Signal 17:1178–1189. doi:10.1089/ars.2011.4226. 22424265

[31] Mesa S, Bedmar EJ, Chanfon A, Hennecke H, Fischer HM. 2003. *Bradyrhizobium japonicum* NnrR, a denitrification regulator, expands the FixLJ-FixK2 regulatory cascade. J Bacteriol 185:3978–3982. doi:10.1128/JB.185.13.3978-3982.2003. 12813094 PMC161565

[32] Laratta WP, Shapleigh JP. 2003. Site-directed mutagenesis of NnrR: a transcriptional regulator of nitrite and nitric oxide reductase in *Rhodobacter sphaeroides*. FEMS Microbiol Lett 229:173–178. doi:10.1016/S0378-1097(03)00821-8. 14680695

[33] Marino M, Ramos HC, Hoffmann T, Glaser P, Jahn D. 2001. Modulation of anaerobic energy metabolism of *Bacillus subtilis* by *arfM* (*ywiD*). J Bacteriol 183:6815–6821. doi:10.1128/JB.183.23.6815-6821.2001. 11698370 PMC95522

[34] Cho KH, Cho D, Wang G-R, Salyers AA. 2001. New regulatory gene that contributes to control of *Bacteroides thetaiotaomicron* starch utilization genes. J Bacteriol 183:7198–7205. doi:10.1128/JB.183.24.7198-7205.2001. 11717279 PMC95569

[35] Doğan T, MacDougall A, Saidi R, Poggioli D, Bateman A, O'Donovan C, Martin MJ. 2016. UniProt-DAAC: domain architecture alignment and classification, a new method for automatic functional annotation in UniProtKB. Bioinformatics 32:2264–2271. doi:10.1093/bioinformatics/btw114. 27153729 PMC4965628

[36] Roberts GP, Kerby RL, Youn H, Conrad M. 2005. CooA, a paradigm for gas sensing regulatory proteins. J Inorg Biochem 99:280–292. doi:10.1016/j.jinorgbio.2004.10.032. 15598507

[37] Kanehisa M, Goto S. 2000. KEGG: Kyoto Encyclopedia of Genes and Genomes. Nucleic Acids Res 28:27–30. doi:10.1093/nar/28.1.27. 10592173 PMC102409

[38] Kolb A, Busby S, Buc II, Garges S, Adhya S. 1993. Transcriptional regulation by cAMP and its receptor protein. Annu Rev Biochem 62:749–797. doi:10.1146/annurev.bi.62.070193.003533. 8394684

[39] Nanchen A, Schicker A, Revelles O, Sauer U. 2008. Cyclic AMP-dependent catabolite repression is the dominant control mechanism of metabolic fluxes under glucose limitation in *Escherichia coli*. J Bacteriol 190:2323–2330. doi:10.1128/JB.01353-07. 18223071 PMC2293195

[40] Gosset G, Zhang Z, Nayyar S, Cuevas WA, Saier MH. Jr., 2004. Transcriptome analysis of Crp-dependent catabolite control of gene expression in *Escherichia coli*. J Bacteriol 186:3516–3524. doi:10.1128/JB.186.11.3516-3524.2004. 15150239 PMC415760

[41] Zheng D, Constantinidou C, Hobman JL, Minchin SD. 2004. Identification of the CRP regulon using in vitro and in vivo transcriptional profiling. Nucleic Acids Res 32:5874–5893. doi:10.1093/nar/gkh908. 15520470 PMC528793

[42] Grainger DC, Hurd D, Harrison M, Holdstock J, Busby SJ. 2005. Studies of the distribution of *Escherichia coli* cAMP-receptor protein and RNA polymerase along the *E. coli* chromosome. Proc Natl Acad Sci USA 102:17693–17698. doi:10.1073/pnas.0506687102. 16301522 PMC1308901

[43] Ravcheev DA, Godzik A, Osterman AL, Rodionov DA. 2013. Polysaccharides utilization in human gut bacterium *Bacteroides thetaiotaomicron*: comparative genomics reconstruction of metabolic and regulatory networks. BMC Genomics 14:873. doi:10.1186/1471-2164-14-873. 24330590 PMC3878776

[44] Rodionov DA, Dubchak IL, Arkin AP, Alm EJ, Gelfand MS. 2005. Dissimilatory metabolism of nitrogen oxides in bacteria: comparative reconstruction of transcriptional networks. PLoS Comput Biol 1:e55. doi:10.1371/journal.pcbi.0010055. 16261196 PMC1274295

[45] Rodionov DA, Dubchak I, Arkin A, Alm E, Gelfand MS. 2004. Reconstruction of regulatory and metabolic pathways in metal-reducing delta-proteobacteria. Genome Biol 5:R90. doi:10.1186/gb-2004-5-11-r90. 15535866 PMC545781

[46] Townsend GE, II, Han W, Schwalm ND, III, Hong X, Bencivenga-Barry NA, Goodman AL, Groisman EA. 2020. A master regulator of *Bacteroides thetaiotaomicron* gut colonization controls carbohydrate utilization and an alternative protein synthesis factor. mBio 11:e03221-19. doi:10.1128/mBio.03221-19. 31992627 PMC6989115

[47] Dufour YS, Kiley PJ, Donohue TJ. 2010. Reconstruction of the core and extended regulons of global transcription factors. PLoS Genet 6:e1001027. doi:10.1371/journal.pgen.1001027. 20661434 PMC2908626

[48] Rompf A, Hungerer C, Hoffmann T, Lindenmeyer M, Romling U, Gross U, Doss MO, Arai H, Igarashi Y, Jahn D. 1998. Regulation of *Pseudomonas aeruginosa hemF* and *hemN* by the dual action of the redox response regulators Anr and Dnr. Mol Microbiol 29:985–997. doi:10.1046/j.1365-2958.1998.00980.x. 9767567

[49] Münch R, Hiller K, Barg H, Heldt D, Linz S, Wingender E, Jahn D. 2003. PRODORIC: prokaryotic database of gene regulation. Nucleic Acids Res 31:266–269. doi:10.1093/nar/gkg037. 12519998 PMC165484

[50] Jewell TN, Karaoz U, Brodie EL, Williams KH, Beller HR. 2016. Metatranscriptomic evidence of pervasive and diverse chemolithoautotrophy relevant to C, S, N and Fe cycling in a shallow alluvial aquifer. ISME J 10:2106–2117. doi:10.1038/ismej.2016.25. 26943628 PMC4989316

[51] Wang Y, Gao H, G FW. 2021. Integrated omics analyses reveal differential gene expression and potential for cooperation between denitrifying polyphosphate and glycogen accumulating organisms. Environ Microbiol 23:3274–3293. doi:10.1111/1462-2920.15486. 33769674

[52] Sedlacek CJ, Giguere AT, Dobie MD, Mellbye BL, Ferrell RV, Woebken D, Sayavedra-Soto LA, Bottomley PJ, Daims H, Wagner M, Pjevac P. 2020. Transcriptomic response of *Nitrosomonas europaea* transitioned from ammonia- to oxygen-limited steady-state growth. mSystems 5:e00562-19. doi:10.1128/mSystems.00562-19. 31937676 PMC6967387

[53] Sayavedra-Soto LA, Hommes NG, Russell SA, Arp DJ. 1996. Induction of ammonia monooxygenase and hydroxylamine oxidoreductase mRNAs by ammonium in *Nitrosomonas europaea*. Mol Microbiol 20:541–548. doi:10.1046/j.1365-2958.1996.5391062.x. 8736533

[54] Wei X, Sayavedra-Soto LA, Arp DJ. 2004. The transcription of the *cbb* operon in *Nitrosomonas europaea*. Microbiology (Reading) 150:1869–1879. doi:10.1099/mic.0.26785-0. 15184573

[55] Dangel AW, Tabita FR. 2015. CbbR, the master regulator for microbial carbon dioxide fixation. J Bacteriol 197:3488–3498. doi:10.1128/JB.00442-15. 26324454 PMC4621087

[56] Galimand M, Gamper M, Zimmermann A, Haas D. 1991. Positive FNR-like control of anaerobic arginine degradation and nitrate respiration in *Pseudomonas aeruginosa*. J Bacteriol 173:1598–1606. doi:10.1128/jb.173.5.1598-1606.1991. 1900277 PMC207308

[57] Trunk K, Benkert B, Quack N, Munch R, Scheer M, Garbe J, Jansch L, Trost M, Wehland J, Buer J, Jahn M, Schobert M, Jahn D. 2010. Anaerobic adaptation in *Pseudomonas aeruginosa*: definition of the Anr and Dnr regulons. Environ Microbiol 12:1719–1733. doi:10.1111/j.1462-2920.2010.02252.x. 20553552

[58] Pitcher RS, Brittain T, Watmough NJ. 2002. Cytochrome *cbb3* oxidase and bacterial microaerobic metabolism. Biochem Soc Trans 30:653–658. doi:10.1042/bst0300653. 12196157

[59] Camejo PY, Oyserman BO, McMahon KD, Noguera DR. 2019. Integrated omic analyses provide evidence that a “*Candidatus* Accumulibacter phosphatis” strain performs denitrification under microaerobic conditions. mSystems 4:e00193-18. doi:10.1128/mSystems.00193-18. 30944872 PMC6446978

[60] Hayashi M, Nakayama Y, Unemoto T. 2001. Recent progress in the Na+-translocating NADH-quinone reductase from the marine *Vibrio alginolyticus*. Biochim Biophys Acta 1505:37–44. doi:10.1016/s0005-2728(00)00275-9. 11248187

[61] Camejo PY, Domingo JS, McMahon KD, Noguera DR. 2017. Genome-enabled insights into the ecophysiology of the comammox bacterium “*Candidatus* Nitrospira nitrosa.” mSystems 2:504. doi:10.1128/mSystems.00059-17. PMC5596200 28905001

[62] Spiro S. 2007. Regulators of bacterial responses to nitric oxide. FEMS Microbiol Rev 31:193–211. doi:10.1111/j.1574-6976.2006.00061.x. 17313521

[63] Derouaux A, Wolf B, Fraipont C, Breukink E, Nguyen-Disteche M, Terrak M. 2008. The monofunctional glycosyltransferase of *Escherichia coli* localizes to the cell division site and interacts with penicillin-binding protein 3, FtsW, and FtsN. J Bacteriol 190:1831–1834. doi:10.1128/JB.01377-07. 18165305 PMC2258671

[64] Schwalm ND, III, Townsend GE, II, Groisman EA. 2016. Multiple signals govern utilization of a polysaccharide in the gut bacterium *Bacteroides thetaiotaomicron*. mBio 7:e01342-16. doi:10.1128/mBio.01342-16. 27729509 PMC5061871

[65] Pyles EA, Lee JC. 1996. Mode of selectivity in cyclic AMP receptor protein-dependent promoters in *Escherichia coli*. Biochemistry 35:1162–1172. doi:10.1021/bi952187q. 8573570

[66] Jia M, Castro-Barros CM, Winkler MKH, Volcke EIP. 2018. Effect of organic matter on the performance and N_2_O emission of a granular sludge anammox reactor. Environ Sci: Water Res Technol 4:1035–1046. doi:10.1039/C8EW00125A.

[67] Chen C, Sun F, Zhang H, Wang J, Shen Y, Liang X. 2016. Evaluation of COD effect on anammox process and microbial communities in the anaerobic baffled reactor (ABR). Bioresour Technol 216:571–578. doi:10.1016/j.biortech.2016.05.115. 27285572

[68] Jin R-C, Yang G-F, Yu J-J, Zheng P. 2012. The inhibition of the Anammox process: a review. Chem Eng J 197:67–79. doi:10.1016/j.cej.2012.05.014.

[69] Lawson CE, Nuijten GHL, de Graaf RM, Jacobson TB, Pabst M, Stevenson DM, Jetten MSM, Noguera DR, McMahon KD, Amador-Noguez D, Lucker S. 2021. Autotrophic and mixotrophic metabolism of an anammox bacterium revealed by in vivo ^13^C and ^2^H metabolic network mapping. ISME J 15:673–687. doi:10.1038/s41396-020-00805-w. 33082573 PMC8027424

[70] Baughn AD, Garforth SJ, Vilcheze C, Jacobs WR, Jr., 2009. An anaerobic-type alpha-ketoglutarate ferredoxin oxidoreductase completes the oxidative tricarboxylic acid cycle of *Mycobacterium tuberculosis*. PLoS Pathog 5:e1000662. doi:10.1371/journal.ppat.1000662. 19936047 PMC2773412

[71] Noriega CE, Lin H-Y, Chen L-L, Williams SB, Stewart V. 2010. Asymmetric cross-regulation between the nitrate-responsive NarX-NarL and NarQ-NarP two-component regulatory systems from *Escherichia coli* K-12. Mol Microbiol 75:394–412. doi:10.1111/j.1365-2958.2009.06987.x. 19968795 PMC3034140

[72] Beauchene NA, Myers KS, Chung D, Park DM, Weisnicht AM, Keleş S, Kiley PJ. 2015. Impact of anaerobiosis on expression of the iron-responsive Fur and RyhB regulons. mBio 6:e01947-15. doi:10.1128/mBio.01947-15. 26670385 PMC4676285

[73] Yeo W-S, Lee J-H, Lee K-C, Roe J-H. 2006. IscR acts as an activator in response to oxidative stress for the *suf* operon encoding Fe-S assembly proteins. Mol Microbiol 61:206–218. doi:10.1111/j.1365-2958.2006.05220.x. 16824106

[74] Giel JL, Rodionov D, Liu M, Blattner FR, Kiley PJ. 2006. IscR-dependent gene expression links iron-sulphur cluster assembly to the control of O2-regulated genes in *Escherichia coli*. Mol Microbiol 60:1058–1075. doi:10.1111/j.1365-2958.2006.05160.x. 16677314

[75] Lawson CE, Harcombe WR, Hatzenpichler R, Lindemann SR, Loffler FE, O'Malley MA, Martin HG, Pfleger BF, Raskin L, Venturelli OS, Weissbrodt DG, Noguera DR, McMahon KD. 2019. Common principles and best practices for engineering microbiomes. Nat Rev Microbiol 17:725–741. doi:10.1038/s41579-019-0255-9. 31548653 PMC8323346

[76] Myers KS, Park DM, Beauchene NA, Kiley PJ. 2015. Defining bacterial regulons using ChIP-seq. Methods (San Diego, Calif) 86:80–88. doi:10.1016/j.ymeth.2015.05.022. PMC4577457 26032817

[77] Bartlett A, O'Malley RC, Huang SC, Galli M, Nery JR, Gallavotti A, Ecker JR. 2017. Mapping genome-wide transcription-factor binding sites using DAP-seq. Nat Protoc 12:1659–1672. doi:10.1038/nprot.2017.055. 28726847 PMC5576341

[78] Nifong A, Nelson A, Johnson C, Bott CB. 2013. Performance of a full-scale sidestream DEMON deammonification installation. Proc Water Env Federation 2013:3686–3709. doi:10.2175/193864713813685700.

[79] American Public Health Association (APHA), American Water Works Association (AWWA), and Water Environment Federation (WEF). 2005. Standard methods for the examination of water and wastewater, 21st ed. American Public Health Association, Washington, DC.

[80] Scarborough MJ, Lawson CE, Hamilton JJ, Donohue TJ, Noguera DR. 2018. Metatranscriptomic and thermodynamic insights into medium-chain fatty acid production using an anaerobic microbiome. mSystems 3:e00221-18. doi:10.1128/mSystems.00221-18. 30505946 PMC6247018

[81] Joshi N, Fass J. Accessed 18 October 2018. Sickle: a sliding-window, adaptive, quality-based trimming tool for FastQ files (version 1.33). https://github.com/najoshi/sickle.

[82] Nurk S, Meleshko D, Korobeynikov A, Pevzner PA. 2017. metaSPAdes: a new versatile metagenomic assembler. Genome Res 27:824–834. doi:10.1101/gr.213959.116. 28298430 PMC5411777

[83] Eren AM, Esen OC, Quince C, Vineis JH, Morrison HG, Sogin ML, Delmont TO. 2015. Anvi'o: an advanced analysis and visualization platform for 'omics data. PeerJ 3:e1319. doi:10.7717/peerj.1319. 26500826 PMC4614810

[84] Bushnell B. Accessed 18 October 2018. BBMap: a fast, accurate, splice-aware aligner. Lawrence Berkeley National Laboratory. LBNL report no. LBNL-7065E. https://escholarship.org/uc/item/1h3515gn.

[85] Li H, Handsaker B, Wysoker A, Fennell T, Ruan J, Homer N, Marth G, Abecasis G, Durbin R, Genome Project Data Processing Subgroup. 2009. The Sequence Alignment/Map format and SAMtools. Bioinformatics 25:2078–2079. doi:10.1093/bioinformatics/btp352. 19505943 PMC2723002

[86] Hyatt D, Chen GL, Locascio PF, Land ML, Larimer FW, Hauser LJ. 2010. Prodigal: prokaryotic gene recognition and translation initiation site identification. BMC Bioinformatics 11:119. doi:10.1186/1471-2105-11-119. 20211023 PMC2848648

[87] Eddy SR. 2011. Accelerated profile HMM searches. PLoS Comput Biol 7:e1002195. doi:10.1371/journal.pcbi.1002195. 22039361 PMC3197634

[88] Galperin MY, Makarova KS, Wolf YI, Koonin EV. 2015. Expanded microbial genome coverage and improved protein family annotation in the COG database. Nucleic Acids Res 43:D261–9. doi:10.1093/nar/gku1223. 25428365 PMC4383993

[89] Alneberg J, Bjarnason BS, de Bruijn I, Schirmer M, Quick J, Ijaz UZ, Lahti L, Loman NJ, Andersson AF, Quince C. 2014. Binning metagenomic contigs by coverage and composition. Nat Methods 11:1144–1146. doi:10.1038/nmeth.3103. 25218180

[90] Parks DH, Chuvochina M, Chaumeil PA, Rinke C, Mussig AJ, Hugenholtz P. 2020. A complete domain-to-species taxonomy for Bacteria and Archaea. Nat Biotechnol 38:1079–1086. doi:10.1038/s41587-020-0501-8. 32341564

[91] Tennessen K, Andersen E, Clingenpeel S, Rinke C, Lundberg DS, Han J, Dangl JL, Ivanova N, Woyke T, Kyrpides N, Pati A. 2016. ProDeGe: a computational protocol for fully automated decontamination of genomes. ISME J 10:269–272. doi:10.1038/ismej.2015.100. 26057843 PMC4681846

[92] Parks DH, Imelfort M, Skennerton CT, Hugenholtz P, Tyson GW. 2015. CheckM: assessing the quality of microbial genomes recovered from isolates, single cells, and metagenomes. Genome Res 25:1043–1055. doi:10.1101/gr.186072.114. 25977477 PMC4484387

[93] Seemann T. 2014. Prokka: rapid prokaryotic genome annotation. Bioinformatics 30:2068–2069. doi:10.1093/bioinformatics/btu153. 24642063

[94] Altschul SF, Gish W, Miller W, Myers EW, Lipman DJ. 1990. Basic local alignment search tool. J Molecular Biology 215:403–410. doi:10.1016/S0022-2836(05)80360-2. 2231712

[95] Camacho C, Coulouris G, Avagyan V, Ma N, Papadopoulos J, Bealer K, Madden TL. 2009. BLAST+: architecture and applications. BMC Bioinformatics 10:421. doi:10.1186/1471-2105-10-421. 20003500 PMC2803857

[96] Laslett D, Canback B. 2004. ARAGORN, a program to detect tRNA genes and tmRNA genes in nucleotide sequences. Nucleic Acids Res 32:11–16. doi:10.1093/nar/gkh152. 14704338 PMC373265

[97] Seemann T. Accessed 18 October 2018. Barrnap 0.9: rapid ribosomal RNA prediction. https://github.com/tseemann/barrnap.

[98] Taboada B, Estrada K, Ciria R, Merino E. 2018. Operon-mapper: a web server for precise operon identification in bacterial and archaeal genomes. Bioinformatics 34:4118–4120. doi:10.1093/bioinformatics/bty496. 29931111 PMC6247939

[99] Andrews S. Accessed 18 October 2018. FastQC: a quality control tool for high throughput sequence data. https://www.bioinformatics.babraham.ac.uk/projects/fastqc/.

[100] Kopylova E, Noé L, Touzet H. 2012. SortMeRNA: fast and accurate filtering of ribosomal RNAs in metatranscriptomic data. Bioinformatics 28:3211–3217. doi:10.1093/bioinformatics/bts611. 23071270

[101] Anders S, Pyl PT, Huber W. 2015. HTSeq—a Python framework to work with high-throughput sequencing data. Bioinformatics 31:166–169. doi:10.1093/bioinformatics/btu638. 25260700 PMC4287950

[102] Bullard JH, Purdom E, Hansen KD, Dudoit S. 2010. Evaluation of statistical methods for normalization and differential expression in mRNA-Seq experiments. BMC Bioinformatics 11:94. doi:10.1186/1471-2105-11-94. 20167110 PMC2838869

[103] Hertz GZ, Stormo GD. 1999. Identifying DNA and protein patterns with statistically significant alignments of multiple sequences. Bioinformatics 15:563–577. doi:10.1093/bioinformatics/15.7.563. 10487864

[104] McDowell IC, Manandhar D, Vockley CM, Schmid AK, Reddy TE, Engelhardt BE. 2018. Clustering gene expression time series data using an infinite Gaussian process mixture model. PLoS Comput Biol 14:e1005896. doi:10.1371/journal.pcbi.1005896. 29337990 PMC5786324

