# Correction for Beach et al., “Exploring the Meta-regulon of the CRP/FNR Family of Global Transcriptional Regulators in a Partial-Nitritation Anammox Microbiome”

**DOI:** 10.1128/msystems.00213-22

**Published:** 2022-04-13

**Authors:** Natalie K. Beach, Kevin S. Myers, Brian R. Owen, Matt Seib, Timothy J. Donohue, Daniel R. Noguera

**Affiliations:** a Department of Civil and Environmental Engineering, University of Wisconsin-Madison, Madison, Wisconsin, USA; b Carollo Engineers, Inc., Broomfield, Colorado, USA; c Great Lakes Bioenergy Research Center, University of Wisconsin-Madison, Madison, Wisconsin, USA; d Wisconsin Energy Institute, University of Wisconsin-Madison, Madison, Wisconsin, USA; e Madison Metropolitan Sewerage District, Madison, Wisconsin, USA; f Department of Bacteriology, University of Wisconsin-Madison, Madison, Wisconsin, USA; g Carollo Engineers, Inc., San Diego, California, USA

## AUTHOR CORRECTION

Volume 6, no. 5, e00906-21, 2021, https://doi.org/10.1128/mSystems.00906-21. Page 19: In the Acknowledgments section, a grant number for the National Science Foundation (NSF) was inadvertently omitted. The correct acknowledgment for NSF funding should include two grant numbers, CBET-1435661 and CBET-1803055.

